# Autonomous Targeting of Infectious Superspreaders Using Engineered Transmissible Therapies

**DOI:** 10.1371/journal.pcbi.1002015

**Published:** 2011-03-17

**Authors:** Vincent T. Metzger, James O. Lloyd-Smith, Leor S. Weinberger

**Affiliations:** 1 Department of Chemistry and Biochemistry, University of California, San Diego, La Jolla, California, United States of America; 2 Department of Ecology and Evolutionary Biology, University of California, Los Angeles, Los Angeles, California, United States of America; 3 Fogarty International Center, National Institutes of Health, Bethesda, Maryland, United States of America; 4 Division of Infectious Diseases, Department of Medicine, University of California, San Diego, La Jolla, California, United States of America; Utrecht University, The Netherlands

## Abstract

Infectious disease treatments, both pharmaceutical and vaccine, face three universal challenges: the difficulty of targeting treatments to high-risk ‘superspreader’ populations who drive the great majority of disease spread, behavioral barriers in the host population (such as poor compliance and risk disinhibition), and the evolution of pathogen resistance. Here, we describe a proposed intervention that would overcome these challenges by capitalizing upon Therapeutic Interfering Particles (TIPs) that are engineered to replicate conditionally in the presence of the pathogen and spread between individuals — analogous to ‘transmissible immunization’ that occurs with live-attenuated vaccines (but without the potential for reversion to virulence). Building on analyses of HIV field data from sub-Saharan Africa, we construct a multi-scale model, beginning at the single-cell level, to predict the effect of TIPs on individual patient viral loads and ultimately population-level disease prevalence. Our results show that a TIP, engineered with properties based on a recent HIV gene-therapy trial, could stably lower HIV/AIDS prevalence by ∼30-fold within 50 years and could complement current therapies. In contrast, optimistic antiretroviral therapy or vaccination campaigns alone could only lower HIV/AIDS prevalence by <2-fold over 50 years. The TIP's efficacy arises from its exploitation of the same risk factors as the pathogen, allowing it to autonomously penetrate superspreader populations, maintain efficacy despite behavioral disinhibition, and limit viral resistance. While demonstrated here for HIV, the TIP concept could apply broadly to many viral infectious diseases and would represent a new paradigm for disease control, away from pathogen eradication but toward robust disease suppression.

## Author Summary

We introduce a proposed intervention against infectious diseases that extends and optimizes the recognized benefit of ‘transmissible immunization’ that occurs with live-attenuated vaccines such as Oral Polio Vaccine (OPV), the vaccine chosen for the worldwide polio eradication campaign. The intervention proposed here is based upon Therapeutic Interfering Particles (TIPs) that are engineered to replicate only in the presence of the wildtype pathogen and act to inhibit the growth of the pathogen. Therefore TIPs ‘piggyback’ on the pathogen, leading to two important differences from live-attenuated vaccines: TIPs can only transmit from individuals already infected with wildtype pathogen, and TIPs could only revert to virulence in individuals already carrying the wild-type pathogen. Intriguingly, because TIPs spread between individuals using the same transmission routes as the pathogen, they automatically find their way to the populations at greatest risk of infection, thus circumventing the unsolved problem of how to identify superspreaders and target them for preventive measures. Based on clinical-trial data, we analyze the impact that TIP intervention would have on HIV/AIDS in sub-Saharan Africa and show that TIPs could lower HIV/AIDS prevalence more effectively than vaccines or drugs alone and, in fact, would effectively complement these other interventions.

## Introduction

From ‘core groups’ to ‘superspreaders’, epidemiologists have long recognized the immense potential of targeting high-risk groups for efficient control of infectious diseases [1], [2], [3], [4]. These groups are often described by the classic ‘80/20 rule’ [5] where 20% of the individuals drive 80% of disease transmission and thus dominate the overall pattern of disease prevalence. For sexually transmitted and blood-borne infections such as Hepatitis C [6], syphilis [7], and HIV-1 [8], [9] (here termed HIV), superspreading is driven by high-risk sexual or needle-sharing behaviors. For many other pathogens, spanning a broad range of transmission modes and life histories, superspreading plays an important role in transmission dynamics but the underlying mechanisms remain poorly understood [4], [10], [11].

Targeting these superspreader subpopulations for therapeutic or preventive measures would tremendously increase the efficacy of disease control [3], [4], while failure to target high-risk groups weakens efforts to achieve ‘herd immunity’ by vaccination and severely limits the ability to reduce disease at the population level [12]. Unfortunately, identifying these crucial high-risk populations requires in-depth knowledge of the social or sexual networks that underlie disease spread, which is rarely attainable [13], as well as knowledge of as-yet unknown biological correlates of risk. Further aggravating the problem of targeting superspreaders are: (i) non-healthseeking behaviors in the key populations, such as injection drug users (IDUs); and (ii) self-concealment motivated by social stigmas and criminal barriers in high-risk individuals, such as IDUs, men who have sex with men, people with extra-marital sexual partners, and commercial sex workers and their clients.

The resulting high cost and effort involved in identifying high-risk populations has meant that—despite the huge potential benefits—targeting of disease control measures to high-risk populations is often not feasible in practice [14]. Here, we propose a fundamentally different approach that obviates the need to directly identify high-risk populations by engineering a therapeutic version of interfering particles (i.e. TIPs) that spread between individuals to autonomously target high-risk groups. The results demonstrate the potential of TIPs to control HIV in sub-Saharan Africa and we benchmark the performance of TIPs against the more familiar alternatives of antiretroviral therapy (ART), which is known to effectively reduce HIV incidence and prevalence, and a hypothetical protective vaccine against HIV. We further demonstrate that the effect of TIPs is complementary to ART programs, so our proposed therapy could be rolled out synergistically with current campaigns.

### The concept: A proposal for transmissible gene therapies

The TIP concept capitalizes upon and extends the phenomenon of interfering particles that occur naturally in many viruses, spread along with the viral pathogen [15], and have demonstrated potential therapeutic efficacy against HIV [16], [17], [18], [19]. TIPs are minimal versions of the pathogen engineered to lack the virulent replication and structural genes of the wild-type pathogen and instead encode therapeutic elements that target key host or viral processes. Since a TIP genome is significantly shorter than the wild-type virus genome, TIP genomes are synthesized at a faster rate, resulting in increased numbers of TIP genomes compared to wild-type virus genomes in the infected cell (see Text S1 and [15]). Specifically, for HIV, the proposed TIP is a lentiviral gene-therapy vector that lacks all structural and envelope genes required to self-replicate, but retains HIV's genomic packaging signals. The TIP can mobilize out of the infected cell only by co-opting wild-type HIV capsid and envelope gene-products [16]. By parasitizing a pathogen's resources, TIPs mobilize from cell to cell [16], [18] and, in a recent clinical trial, this mobilization of a gene-therapy vector against HIV did not appear to be detrimental to patient health [17]. Due to their ability to mobilize and reproduce within hosts, TIPs have the potential to decrease wild-type pathogen levels *in vivo* by many orders of magnitude [19].

By sharing all packaging elements with the wild-type pathogen, TIPs also have the potential to spread between individuals [20], and would spread via the same transmission routes as the disease-causing pathogen. In this respect, combating an infectious disease using TIPs raises unique safety and ethical concerns but bears similarity to the use of live attenuated vaccines. In particular, a recognized advantage of Oral Polio Vaccine (OPV) is that it replicates *in vivo* and sheds, thereby transmitting among susceptible hosts and delivering additional protection via ‘transmissible immunization’ at the population scale [21]. There are, however, crucial differences between TIPs and live attenuated vaccines: (i) TIPs cannot replicate in uninfected hosts and, at most, the TIP will remain dormant until the host is coinfected by wildtype pathogen [22]; and (ii) replication elements are missing from the TIP, so, unlike OPV, TIP cannot revert to virulence in healthy individuals.

## Results/Discussion

### Projected impact of TIPs as HIV control measures

**Figure 1:**
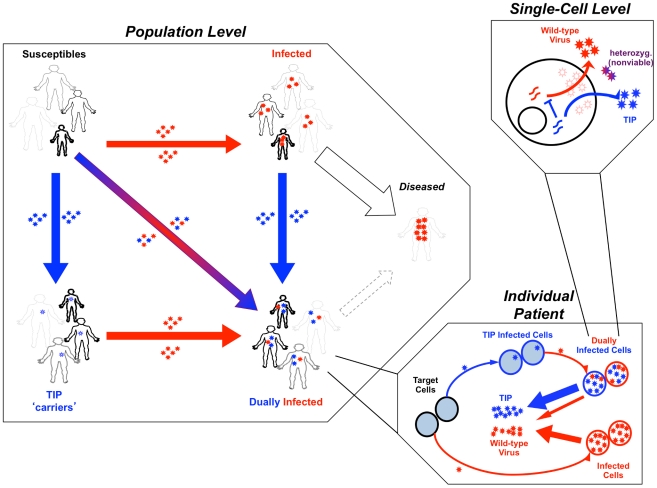
Therapeutic Interfering Particle (TIP) intervention modeled at multiple scales. (*Upper right box*) Schematic of the sub-cellular level model where TIP genomes (blue) mobilize by ‘stealing’ packaging elements from the wild-type virus (red, e.g. HIV) within a dually infected cell [22]. (*Lower right box*) Schematic of the *in vivo* model where TIP (blue) is produced from dually infected cells and reduces wild-type HIV viral set-point [19] within a dually infected individual. (*Left box*) Schematic of the population model where TIP and HIV transmit between individuals of different sexual activity classes (based on UNAIDS Malawi antenatal clinic data [12]). Boldness of figures represents transmission rate, size of figures represents size of sexual activity class. Smallest but boldest figures represent the superspreaders (the least in number but the highest transmission rate). Largest but lightest figures represent individuals with the lowest transmission rates (the greatest in number). Infection by TIP alone (blue) converts susceptible individuals to into latent ‘carriers’ of integrated TIP genomes [22]. Infection by HIV converts susceptible individuals to individuals who progress to disease in ∼10 yrs. Dual infection generates individuals who progress to disease more slowly. Disease progression and transmission rates are proportional to *in vivo* viral loads [25].

To test whether a TIP against HIV could autonomously target high-risk groups, and effectively reduce HIV prevalence, we build upon an established epidemiological model of HIV/AIDS transmission in sub-Saharan Africa that includes four classes of sexual risk behavior based on field data [12]. We develop a data-driven, three-scale model (Figure 1) that translates molecular-level characteristics of the TIP to predict patient-level HIV viral load and ultimately predict HIV/AIDS incidence and prevalence at the population scale. At the single-cell level, the model considers the dynamics of competition between TIP genomic mRNA and HIV genomic mRNA for packaging components [23]. These molecular-level effects of the TIP are translated to viral loads using an established *in vivo* model of HIV dynamics [24] that includes TIP dynamics [19]. Measured relationships between viral load and transmission [25] are used to estimate TIP and HIV transmission rates between individuals, and the rate of disease progression is estimated based on field data of HIV viral load [26] (see Text S1).

For ART, the model assumes an optimistic ‘test-and-treat’ deployment [27] where 75% of all infections in both high-risk and low-risk populations are treated with regimens that stop 99% of all HIV transmission [27]. Our test-and-treat model differs from some previous projections [27], [28] by incorporating two additional behavioral factors described in real populations [29]: (i) ART failure or dropout rates that have been measured in sub-Saharan African populations [30], [31], [32]; (ii) population risk structure. While our model predicts smaller benefits from test-and-treat programs than some earlier work [27], [28], the results are consistent with previous ART projections that have incorporated risk structure [12], [33].

For the vaccine, the model assumes optimistic immunization coverage (80% or 95% coverage) of both high-risk and low-risk populations and considers a vaccine that is 30% protective, slightly higher than reported in the recent ‘Thai trial’ [34], or a hypothetical 50% protective vaccine; life-long efficacy is assumed for both vaccines (i.e. no HIV mutational escape) but not for the TIP. For the TIPs, we analyze interventions that generate a 0.5-Log to 1.5-Log viral-load reduction *in vivo*, as reported in a recent HIV gene-therapy trial [17]. The model predicts the effects of vaccination or TIP intervention on HIV/AIDS prevalence in a resource-poor sub-Saharan setting.

**Figure 2:**
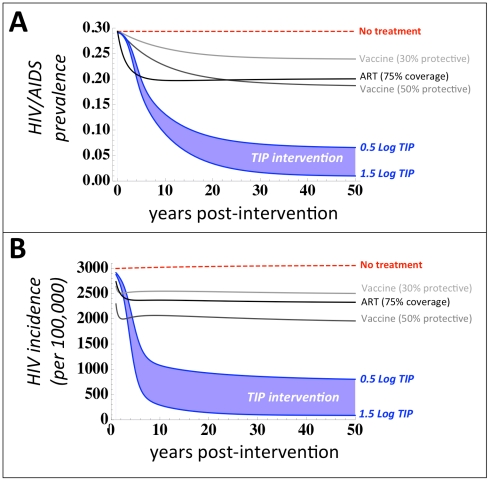
TIPs out-perform optimistic HIV vaccines and antiretroviral therapy (ART). Projected impact of TIP intervention on (*a*) HIV/AIDS disease prevalence over 50 years (*b*) and HIV incidence per 100,000 individuals over 30 years, for two scenarios of TIP efficacy: a 0.5-Log viral-load reduction (upper blue line) and a 1.5-Log viral load reduction (lower blue line), based on a recent clinical trial [17], both initially deployed to 1% of individuals. TIP intervention is compared to a 30% protective vaccine (light grey), a 50% protective vaccine (dark grey), and ART (black). Vaccine scenarios are based on protection levels reported in a recent clinical trial [34] and UNAIDS target protection goals (50% protection) where each vaccine is assumed to have lifelong efficacy and optimistic levels of coverage (80% and 95% coverage of all risk groups, respectively). The ART scenario is assumed to treat 75% of all infections using a universal test-and-treat approach [27] where ART has 99% efficacy in halting HIV transmission.

Strikingly, TIP intervention reduces disease prevalence and incidence more effectively than either widespread ART or a 30% or 50% protective vaccine against HIV/AIDS (Figure 2a–b). The least effective TIP analyzed—which reduces HIV *in vivo* viral load by 0.5-Log (from 10^5^ to 10^4.5^ copies/mL)—leads to a reduction in HIV/AIDS prevalence from 29% to 6.5% in 50 years, despite initial deployment to only 1% of individuals while a TIP that generates a 1.5-Log decrease in HIV viral-load—as transiently achieved in a Phase-I clinical trial for an HIV gene-therapy [17]—would reduce HIV/AIDS prevalence from 29% to below 1% prevalence in 30 years (Figure 2a). In comparison, a 30% protective vaccine deployed to 80% of the entire population (including 8 out of 10 uninfected high-risk individuals) reduces HIV/AIDS prevalence from 29% to 23.9% in 50 years and a 50% protective vaccine deployed to 95% of the entire population (including virtually all uninfected high-risk individuals) reduces HIV/AIDS prevalence from 29% to 18.7% in 50 years. ART to treat 75% of all new infections would reduce disease prevalence to a level between a 30% and 50% protective vaccine. A striking short-term impact of TIP intervention on HIV incidence, as compared to vaccines and ART, is also projected (Figure 2b) despite extremely rapid rollout of vaccines and ART (Figure S1 in Text S1). Similar results are obtained when comparing TIP intervention to vaccination and ART in terms of either the fraction-of-individuals-living-with-AIDS or AIDS incidence (Figure S2 in Text S1). Thus, TIPs constructed using parameters recently reported in Phase-I trials [17], and given to a small fraction of the population (1%), have the potential to swiftly and substantially reduce disease burden at the population level.

This efficacy and robustness of TIP intervention arises from the unique and defining ability of TIPs to transmit between hosts. Analysis of TIPs that generate a 0.5–1.5 Log decrease in viral load, but do not transmit between hosts, shows only a minimal decrease in population-level disease burden (Figure S3 in Text S1)—in agreement with the projected impact of acyclovir treatment which also generates a ∼0.5 Log decrease in HIV viral load [33]. Accordingly, we have paid particular attention to ensuring that our results are robust with respect to changes in basic model assumptions about transmission biology and robust under parameter sensitivity analysis (see Text S1). We also consider two competing models of HIV transmission biology—infection by either a single ‘founder’ virus that enters the new host individual or ‘bottlenecking’ where multiple viruses enter and replicate locally but are then winnowed down by competition within the host [35], [36], [37]—and we provide arguments that our treatment of TIP transmission is consistent with either transmission mode and that TIPs could transmit efficiently in either case (see Text S1 section entitled: “*Considerations for TIP transmission to uninfected hosts*”). To be completely sure that our model results are robust to changes in assumptions about TIP transmission, we repeated the simulations under the worst-case assumption that TIPs are completely unable to transmit in the absence of HIV, and found results that are qualitatively unchanged from Figure 2 (see Text S1 section: “*Sensitivity of model to removal of independent transmission of TIPs (i.e. removal of S_T_ individuals)*”). This somewhat surprising result arises because TIPs autonomously target the highest-risk groups, which are highly likely to be already infected with HIV due to their high-risk status, and thus the majority of the TIP infection ‘flow’ occurs through the already infected individuals. In summary, while there is physiological basis to support that TIPs could transmit efficiently to HIV-uninfected persons, the efficacy of TIP intervention is largely independent of this assumption (i.e. TIPs need not convert susceptible individuals into ‘TIP carriers’ for population-level efficacy to be retained).

These results are not intended to argue that ART campaigns be abandoned or vaccine trials be halted. On the contrary, as we show below, the TIP's ability to target high-risk groups allows the TIP to complement ART (or vaccine) campaigns and significantly enhance the population-level efficacy of these approaches.

### TIPs would circumvent behavioral barriers and complement pharmaceutical treatment

**Figure 3:**
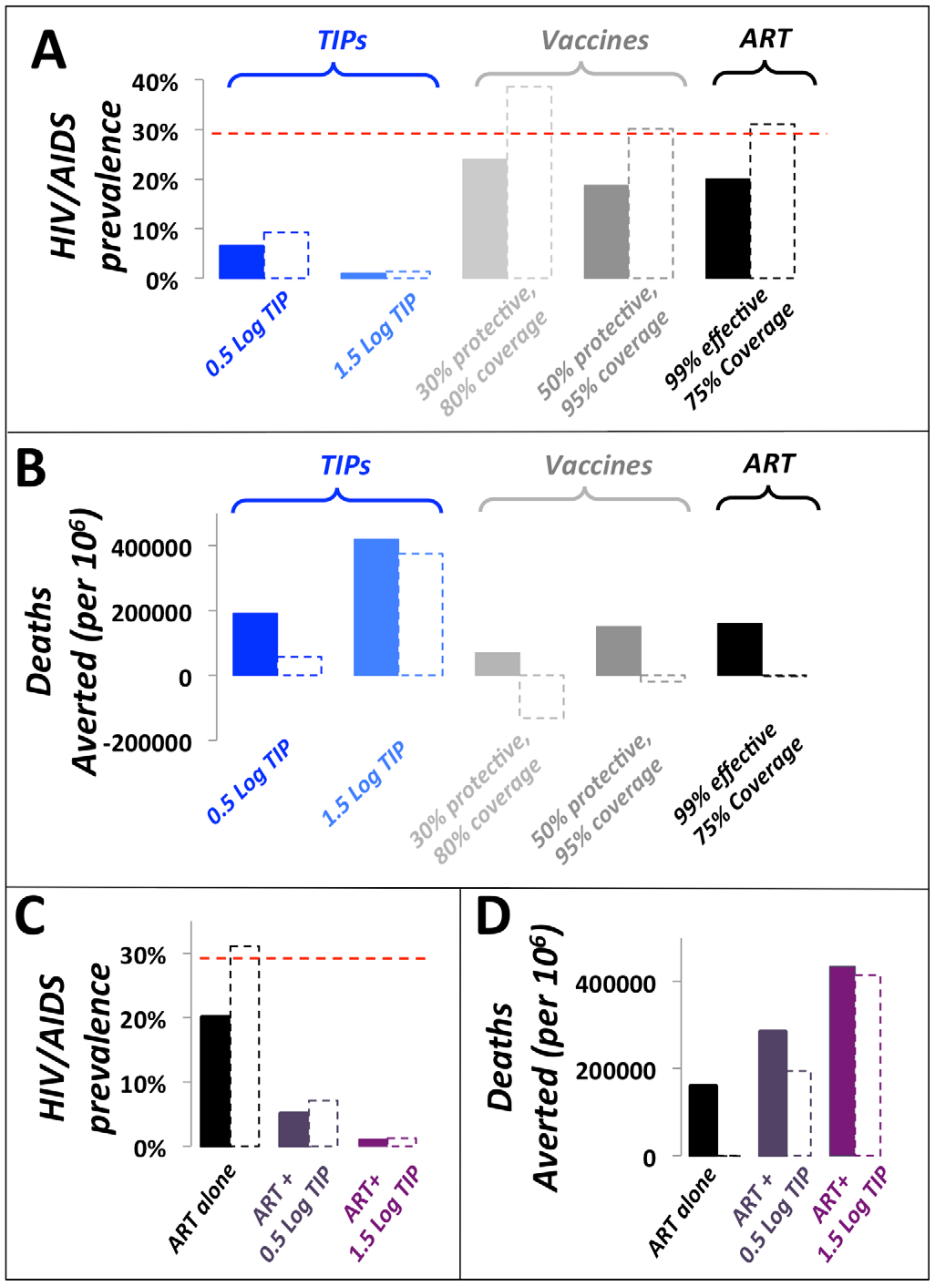
TIPs are resistant to behavioral disinhibition and would complement pharmaceutical approaches to reduce HIV/AIDS disease prevalence. Projected 50-year impact of TIP intervention on (*a*) HIV/AIDS disease prevalence and (*b*) number of deaths averted in the presence and absence of behavioral disinhibition (dashed and solid lines, respectively). TIP interventions (light and dark blue) are compared to vaccination (light and dark grey), ART (black), and the scenario of no intervention (dashed red line). Disinhibition is modeled as in [39] by assuming that individual person-to-person contact rates increase upon introduction of vaccine or TIP intervention. Projected 50-year impact of ART in presence of TIP intervention (*light and dark purple*) on (*c*) HIV/AIDS disease prevalence and (*d*) number of deaths averted compared to projected 50-year impact of ART alone (*black*, *7*5% ART coverage without TIP intervention). Dashed and solid lines are the presence and absence of behavioral disinhibition, respectively. ART and vaccine campaigns are modeled as in Figure 2.

Current prevention and treatment approaches also face the challenges of poor compliance and behavioral disinhibition, wherein successful disease control leads to a reduced sense of personal risk from the disease and can result in increases in risk behavior. Disinhibition is a significant concern for current HIV prevention and control [38] and has the potential to generate the perverse outcome that a successful therapeutic may actually increase HIV incidence [39]. The transmissibility and single-dose administration of TIPs effectively circumvent these problems, unlike current pharmaceutical approaches (i.e. ART) or vaccination. Indeed, the public health benefits of TIPs are uniquely robust to disinhibition, since the intervention spreads more effectively if contact rates increase (Figure 3a). In contrast, the same degree of disinhibition in the presence of ART or a 30% or 50% protective vaccine could have the unfortunate effect of increasing HIV/AIDS prevalence and could increase the number of deaths due to AIDS (Figure 3b), as highlighted by previous analyses [39].

Any intervention against HIV is likely to be administered in the context of the existing ‘standard of care’: ART. Since ART halts HIV transmission, ART would also halt TIP transmission from an individual, leading to the potential that the TIP intervention could be severely hampered. However, the TIP's ability to concentrate in highest-risk groups (see next paragraph), where ART is at best the target coverage fraction (e.g. 75%), would allow TIP intervention to maintain efficacy, reduce HIV/AIDS disease prevalence, and reduce AIDS deaths, more effectively than ART alone, even under optimistic coverage scenarios for ART campaigns (Figure 3c–d). Thus, ART would not interfere with TIP intervention at the population scale, and TIPs could be used as a powerful complement to ART and pharmaceutical treatments in general.

### TIPs would autonomously target high-risk groups

**Figure 4:**
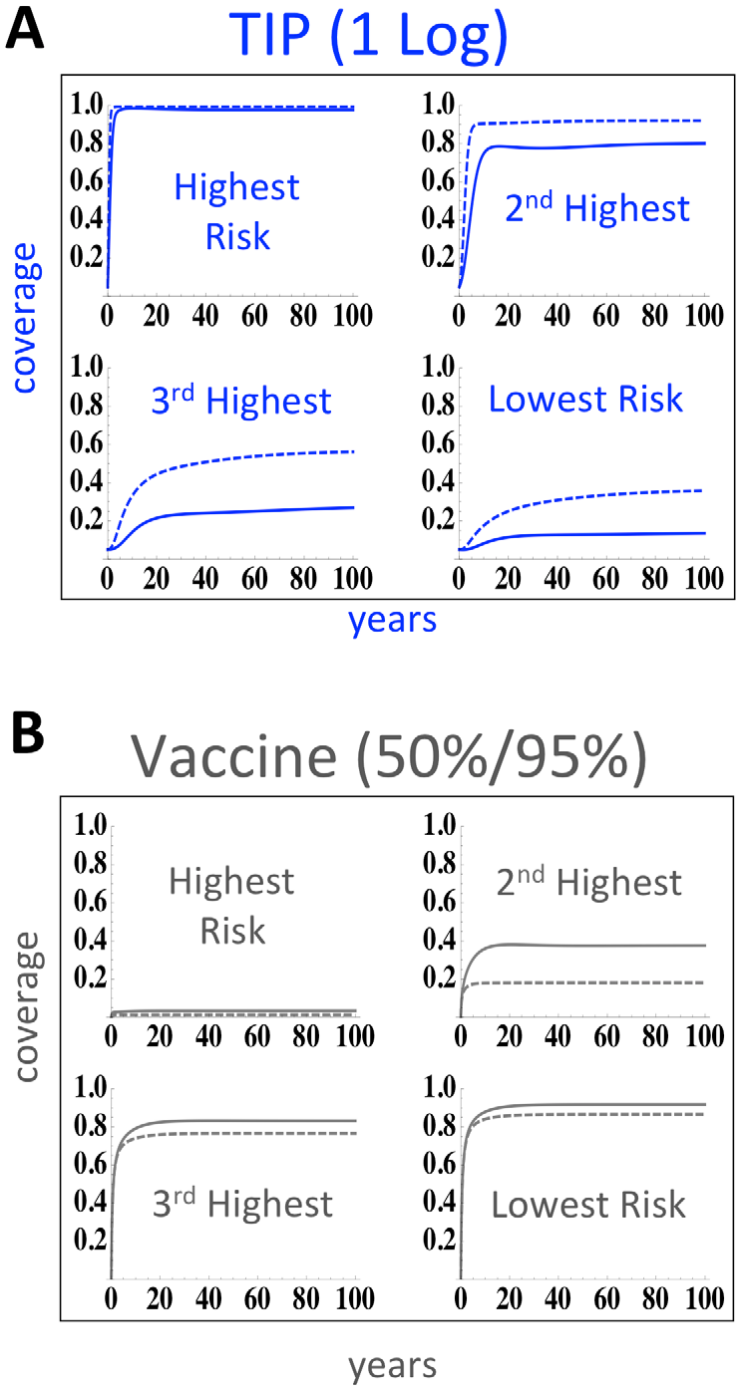
TIPs autonomously target superspreaders. (*a*) Ability of TIP intervention to penetrate each risk-class as reported by fraction of each risk-class exposed to intervention over time. Despite introduction into far more individuals in the lowest risk class (due to our assumption of uniform 1% initial coverage), TIPs can mobilize into the highest risk superspreader class. Solid and dashed lines represent simulations in the presence of behavioral disinhibition, respectively. (*b*) Ability of 50% protective vaccine, administered to 95% of the population, to penetrate each risk-class as reported by fraction of each risk-class exposed to vaccination over time. Solid and dashed lines represent simulations in the presence of behavioral disinhibition, respectively. In contrast to TIPs, a 50% protective vaccine that is directly targeted to the highest risk class is quickly depleted from the highest risk-class because, given partial protection, high-risk individuals still become infected relatively rapidly.

The increased efficacy of TIPs relative to vaccination is due to the TIP's transmissibility along the same transmission routes as the pathogen. Consequently, the TIP transmits to a specific risk group in proportion to that group's risk behavior, leading to more focused targeting of TIPs in more heterogeneous populations and resulting in TIPs concentrating in the highest-risk populations (Figure 4a). In contrast, reaching high-risk classes with ART depends upon active and sustained targeting of these rare high-risk individuals, while partially-protective vaccines tend to concentrate in the lowest risk classes (because higher-risk individuals still become HIV-infected, given partially protective vaccines) and lack the ability to dynamically redistribute between risk classes (Figure 4b).

### Evolutionary considerations

Vaccines and drug treatment strategies also face the challenge of mutation and the strong selective pressure for the pathogen to escape any successful control. For HIV, rapid mutation leads to resistance against anti-retroviral therapy and poses significant challenges for vaccine development [40]. However, unlike conventional therapies, TIPs replicate with the same speed and mutation rate as the pathogen, which sets up an evolutionary arms race between the TIP and the pathogen.

**Figure 5:**
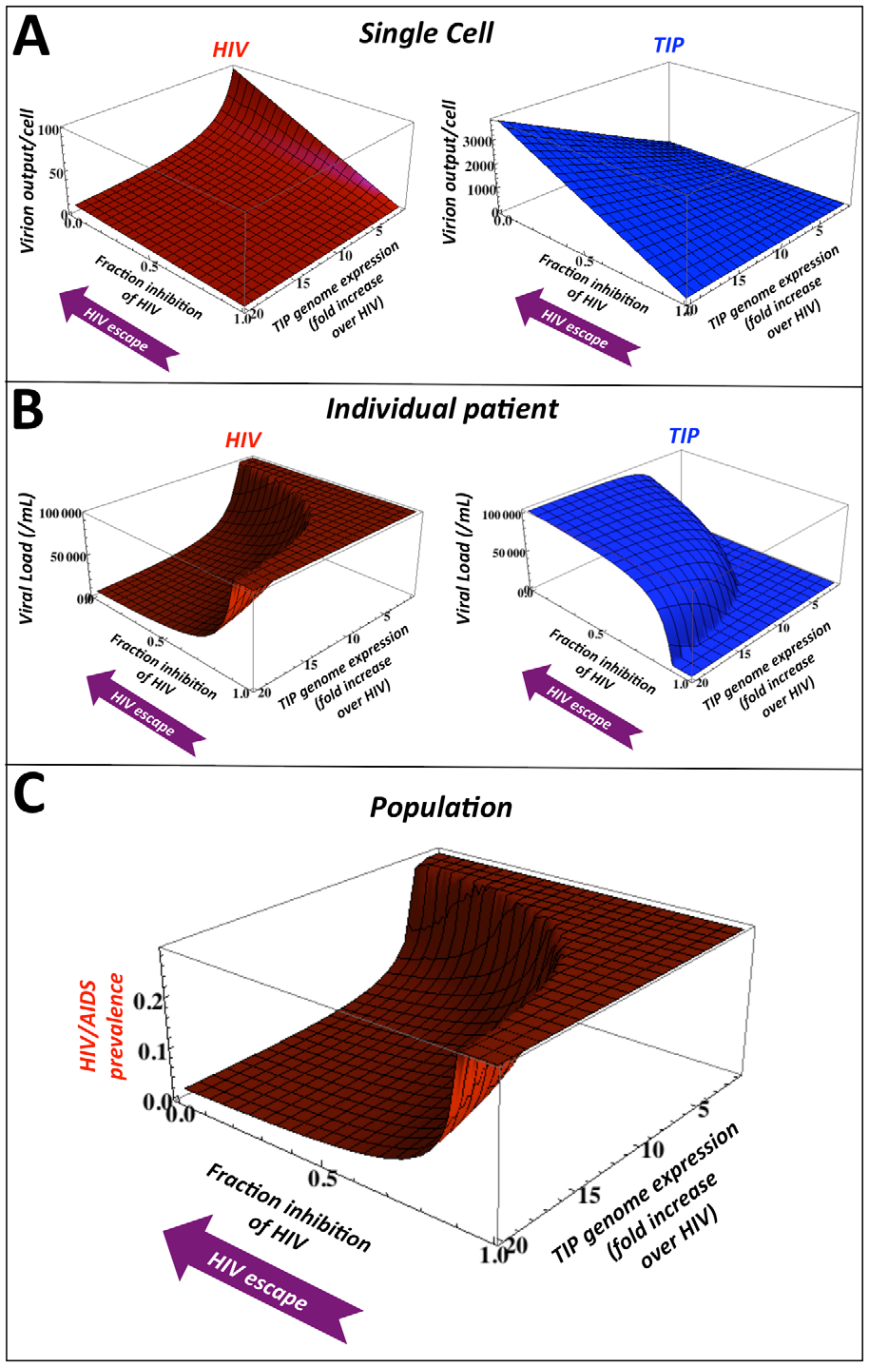
TIP intervention is robust to the evolution of pathogen resistance. Projected steady-state values for: (*a*) HIV and TIP production from dually infected cells at the single-cell level; (*b*) HIV and TIP viral loads at the individual patient level *in vivo*; and (*c*) HIV/AIDS prevalence at the population level. At each scale, values are plotted as a function of two molecular-level design criteria: (i) the expression-level of TIP genomic mRNA over HIV genomic mRNA (parameter *P* from the intracellular model, see Text S1), and (ii) TIP-encoded inhibition of HIV gene expression (parameter *D* from the intracellular model, see Text S1) where 1.0 corresponds to complete inhibition of HIV, 0.0 corresponds to no inhibition of HIV (when *D* = 0.0, HIV viral load is reduced only by ‘wasting’ of HIV genomes in nonviable heterozygous virions). TIPs lacking active inhibition of HIV display higher production at the single-cell level and, counter-intuitively, inhibit HIV more potently at the individual patient level and at the population level by outcompeting HIV for targets. Purple ‘HIV escape’ arrows represent the direction of HIV evolution to evade direct inhibition by TIP-encoded molecules.

To examine how HIV might respond in such an arms race resulting from TIP intervention, we consider the multi-scale dynamics across a range of parameter values for the molecular-level properties used to design a TIP. Specifically, we consider the interplay of HIV and TIP levels as a function of both the strength of TIP-encoded inhibition of HIV and the engineered TIP genomic abundance within a dually infected cell. For HIV, the TIP design encodes an inherent evolutionary tradeoff that generates conflicting selection pressures at different scales (Figure 5). On the one hand, inhibition of HIV replication by TIP-encoded therapy genes inevitably limits TIP production—since any TIP-encoded antiviral that inhibits HIV will compromise the TIP's ability to mobilize. However, due to the diploid nature of retroviral genomes, high concentrations of TIP genomic mRNA alone will inhibit HIV production by wasting the majority of HIV genomes in virions containing one HIV RNA and one TIP RNA, and these heterozygous-diploid virions are not viable [18], [23]. Thus, the lowest TIP-mediated inhibition generates the highest production of TIPs from an infected cell (Figure 5a). The increased numbers of TIP virions then compete more effectively against HIV for target cells which generates a greater reduction in HIV viral-load at the patient-level (Figure 5b), and the lowest HIV/AIDS prevalence in the population (Figure 5c). These results suggest a non-intuitive design criterion for a TIP against HIV: TIPs lacking an inhibitory factor for HIV will be most effective in reducing HIV levels, both in individual patients and at the population level. Similarly, the cellular-scale selective pressure for HIV to escape from TIP-encoded inhibition would point in the same direction (toward zero TIP inhibitory effect) and would lead to increased TIP production (Figure 5a). So, counter-intuitively, HIV escape from TIP-mediated inhibition (at the molecular scale within cells) would reduce HIV viral load and HIV population prevalence to lower levels (Figure 5b–c).

### Safety and ethical considerations

**Figure 6:**
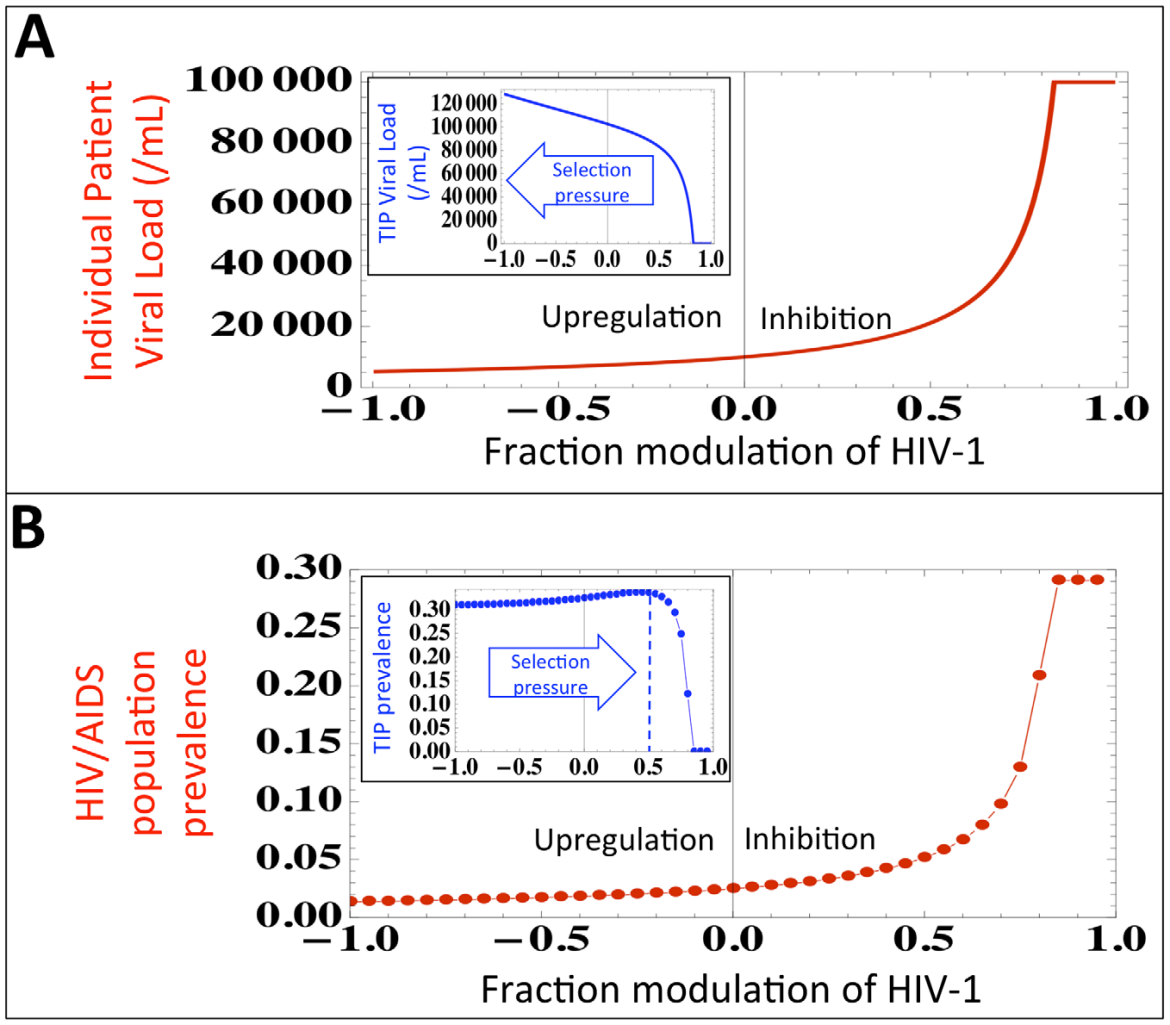
TIPs evolve toward robust reduction in disease prevalence. (*a*) Predicted HIV viral set point as a function of TIP encoded inhibition of HIV (parameter *D* from the intracellular model, see Text S1) where negative inhibition values indicate TIP evolving to upregulate HIV gene expression within a single cell. Inset: Increasing upregulation generates higher TIP viral loads at the individual patient level and leads to lower HIV viral loads. (*b*) HIV/AIDS disease prevalence as a function of TIP encoded inhibition of HIV where negative inhibition values indicate TIP evolving to upregulate HIV expression. Increasing upregulation generates lower HIV/AIDS prevalence at the population level. Inset: TIP prevalence is reduced as upregulation increases, potentially creating an evolutionary trap for the TIP.

The TIP approach carries unique safety concerns [41] and ethical concerns associated with introducing an intervention that transmits and evolves, even in the TIP's limited fashion, within the population. Importantly, clear ethical precedents for transmissible therapies exist in the use of live-attenuated vaccines. Regarding safety, one major concern is that the TIP may recombine with (i.e. acquire) an element that ‘upregulates’ pathogen production and in turn upregulates its own production from the cell. To explore this concern, we examine HIV viral load and population prevalence in the regime where TIP encodes HIV inhibition and in the regime where TIP encodes potential upregulation of HIV gene expression within a single cell. (Figure 6a). As expected, at the single-cell level upregulation of HIV generates increased HIV and TIP production. However, at the individual patient level upregulation of HIV leads to increased TIP viral loads (Figure 6a, inset) which actually generate even lower HIV viral loads (Figure 6a) and HIV population prevalence (Figure 6b). Interestingly, at the population level, there is an optimal value of TIP-encoded inhibition, which yields a maximum in TIP prevalence (Figure 6b, inset). Thus, the TIP appears to be subject to competing selection pressures at multiple scales which may limit the potential for evolutionary breakdown of TIP therapies, echoing recent proposals for antivirals that resist viral escape [42] and ‘evolution-proof’ malaria insecticides [43].

Detailed experimental and theoretical study is required to predict the ultimate direction of TIP evolution, but the competing selection pressures may effectively constrain TIP phenotypes to a range that assures low HIV viral load and low HIV disease prevalence. TIP evolution is likely to be dominated by mutational processes, since recombination between TIPs and wild-type HIV appears to be severely limited by fundamental sequence-homology constraints on retroviral recombination [44] that render recombination between full-length 9.7 kb HIV genomes and shorter lentiviral genomes (e.g. TIP) non-competent for integration [18]. This molecular argument against recombination between HIV and TIP is also supported by data from murine models [45], [46] and the recent human clinical trial data [17], neither of which detected recombination between wild-type HIV-1 and shorter lentiviral therapy vectors.

To fully address safety, there is obviously a need for cautious trials *in vitro*, and *in vivo*, before a TIP intervention could ever be considered for implementation. Importantly, TIPs for HIV would not specifically target, or require, stem cells since the TIP would target the same cells as HIV (primarily CD4^+^ T lymphocytes) and thus oncogenic concerns as a result of insertional mutagenesis [47] are minimized. This argument is supported by a recent Phase-I lentiviral gene-therapy clinical trial for HIV [17] and previous gene therapy in peripheral blood lymphocytes in patients followed since 1995 [48], neither of which detected insertional mutagenesis or oncogenic transformation in patients.

### Conclusion and the way forward

As with all models, our analysis is a relatively simple representation of a complex system and necessarily makes certain assumptions. Importantly, the TIP's robustness and efficacy stems from the unique and defining ability of TIPs to transmit between hosts and, as such, the general results presented for the TIP are qualitatively robust to changes in parameter values or in basic model assumptions about transmission biology (see Text S1). TIP efficacy also appears qualitatively robust to decreases in transmission efficiency as a result of widespread ART coverage (Figure 3c–d) or re-parameterization of transmission functions (see Text S1). Nevertheless, our analysis is intended as a first step towards motivating research into transmissible therapies, rather than a proof of efficacy. The molecular, epidemiological, and ethical bases of using TIP intervention against pathogens will require extensive study, but our results show that TIPs may offer a unique strategy for targeting both high-risk and hard-to-reach populations, overcoming behavioral barriers, and circumventing mutational escape to achieve indefinite disease suppression of HIV, and possibly other pathogens, in resource-limited settings.

As an added benefit for intervention in resource-limited settings, TIPs may have the potential to be administered as a therapy requiring only a single dose, thereby allowing for increased treatment access and minimizing treatment compliance issues. Our results shows that deploying TIPs as a therapy to even a few individuals who are already infected can reduce the prevalence of a disease to very low levels. Due to the rapid and sustained transmission dynamics in high-risk groups, the impact of TIP intervention is robust even if the TIP is quickly cleared from TIP ‘carriers’ so that these individuals rapidly revert back to ‘susceptibles’ (see Text S1). With the ability to enter proviral latency, dormant TIPs could be complementary to ART on an individual scale, by reactivating during ART failure and acting to reduce viral load. While recent models argue that widespread ART campaigns alone could halt the HIV/AIDS pandemic [27], [28], there remains significant controversy as to whether ART can succeed in reducing overall HIV transmission [12], [29], especially in the presence of high-risk groups exhibiting treatment non-compliance. Significant challenges to achieving widespread ART coverage in resource-limited settings include: slower-than-hoped rollout, persistent logistical problems linked to insufficient health systems and weak infrastructure, the need for on-going high-level donor funding, and the social stigmas that prevent people from getting tested and hence starting treatment. These factors will likely produce long-term heterogeneity in coverage, with the most impoverished and disadvantaged groups receiving the least access to ART. Based on these challenges, it is prudent to consider alternative and complementary approaches.

## Materials and Methods

The multi-scale analysis of TIPs and HIV-1 is built upon previous data-driven models [12], [19] and is composed of three constituent ordinary differential equation models describing dynamics at different hierarchical scales: (i) among a population of host individuals (‘population level’) (ii) within host individuals (‘individual patient’) (iii) within infected host cells (‘intracellular’). The multi-scale model specifies mechanistic links between each scale and the next scale of organizational complexity (intracellular → in vivo → population level).

The population-level TIP model is a simplified version of a risk-structured model constructed from UNAIDS field-data collected from antenatal clinics in Malawi [12], which includes a risk-structure formulation with four distinct sexual-activity classes (SACs) and which we refer to as the ‘Baggaley model’. Individuals are classified as susceptible (*S*), HIV infected (*I*), susceptible to HIV but infected with TIP (*S_t_*), dually infected with HIV and TIP (*I_d_*), as an AIDS patient with wild-type virus (*A_w_*), or as a dually infected AIDS patient (*A_d_*). Individuals in all disease-states are divided into SACs in accordance with field data (indicated by subscript *i*), except that all individuals in the *A_w_* class are assumed (as in [12]) to have sexual contacts at the rate corresponding to the lowest risk group (SAC 4) owing to their poor health. The model equations are as follows: 
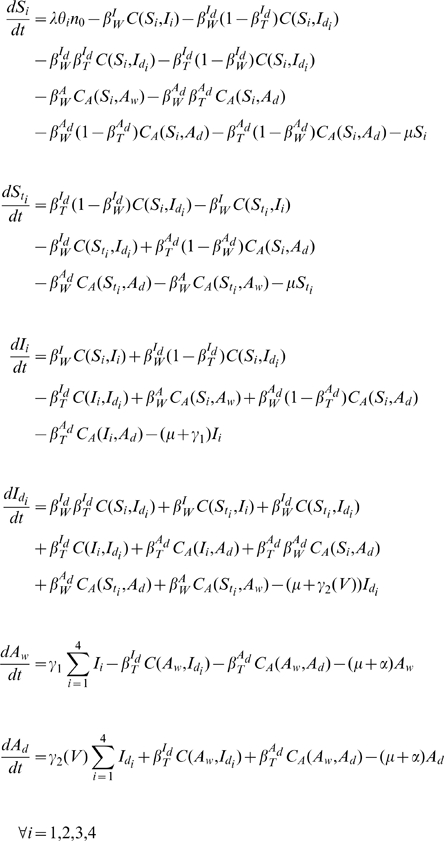



Parameter definitions, values, and corresponding references are shown in Table S1 in Text S1. The transmission probabilities per partnership are denoted 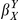 where Y represents the disease state of the source of the infection, and X represents the viral strain (which is wild-type HIV, denoted X = W, for the vaccine model). The per-partnership transmission probability 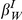 (describing transmission of wild-type HIV by individuals in the *I* disease state) is set to agree with the weighted average of the Baggaley model [12] and 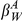 is the per-partnership probability of wild-type HIV infection originating from an AIDS patient, and is set following the Baggaley model [12]. Consideration of alternative parameterizations of the viral load transmission curve did not qualitatively affect the results (see Text S1). The parameters 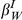 and 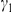 are static parameters that represent the transmission probability and the duration of the asymptomatic phase of individuals infected with only wild-type virus. In contrast, to describe quantities that depend on the specific design of the TIP, such as: (i) transmission probabilities, and (ii) the duration of the asymptomatic period, functions are used in place of parameters. These functions are calculated based on measured correlations between transmission, disease progression, and viral load [25], [26] where viral load is predicted from the *in vivo* TIP model (see Text S1). For example, the transmission probabilities in the presence of TIP and the duration of the asymptomatic phase in dually-infected individuals in the TIP population models are represented by functions of steady-state viral load (i.e. viral set point) as predicted by the *in vivo* model (see Table S3 in Text S1 for a description of the transmission-probability functions). The function 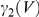 is used to compute the duration of the asymptomatic phase in dually-infected individuals, and is also calculated in Text S1.

Contacts between individuals in the TIP population model are weighted by statistically independent transmission probabilities (*β*) which are calculated from steady-state HIV and TIP viral loads from the *in vivo* model (see Text S1 section: ‘Calculation of Transmission-Rate Function’). There are six distinct transitions between infection classes in the TIP population model (see Table S3 in Text S1 for details). Briefly, contact between two individuals is represented by a contact function that considers asymmetric mixing of individuals among the four SACs:
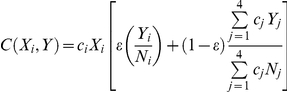



This contact function describes an individual in disease state X (and SAC *i*) becoming infected by an individual in disease state Y. The subscript *j* denotes SAC *j*, *c_j_* is the average number of sexual partners per year in SAC *j*, and *N_j_* is the sum of all sexually active individuals in SAC *j*. In the contact function, 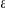 is the degree of assortative mixing with 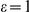 corresponding to entirely assortative mixing and 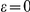 corresponding to entirely random mixing. The first term inside the brackets of the contact function describes assortative mixing in which infected individuals are encountered in proportion to their prevalence in SAC *i*. The second term describes random contacts in which infected individuals are encountered in proportion to their contribution to all of the sexual contacts being made in the entire population. We set the mixing parameter ε equal to 0.37, as estimated in [12].

Simulation of the TIP population model is conducted as follows: the Baggaley model is allowed to reach steady-state and then a TIP is introduced to 1% of all individuals without any targeting to high-risk classes. Similar benefits were obtained using much more restrictive initial conditions (e.g. utilizing TIP as a therapy and targeting TIP to <1% of only *I* and *A_w_* individuals in the least active SACs—SAC 3 and SAC 4—generates similar results to Figure 2). Behavioral disinhibition is simulated as in [49] by increasing the contact rates *c* for all SACs and number of AIDS deaths averted by the vaccination campaign is defined as:

AIDS deaths averted  =  (AIDS deaths during 100 years of epidemic without treatment) – (AIDS deaths during a 50 year epidemic followed by 50 years of treatment).

Vaccine and ART models use the same risk structure as above and are presented in Text S1. A complete list of model parameters and state variables are presented in Tables S1, S2, S3, S4, S5, S6, and S7 in Text S1.

All numerical simulations were performed in Mathematica 7.0.

## Supporting Information

Text S1 Full description of the multi-scale model used to predict the effects of a TIP intervention on HIV-1 infection dynamics among a population of host individuals, within host individuals, and within host cells. This report contains detailed descriptions of each model, tables of parameters and state variables, supporting figures, an analysis of the sensitivity of the TIP model to changes in parameters, and an analysis of the sensitivity of the TIP model to changes in structure and changes in basic transmission biology.
(PDF)
