# Supplementary material for: Autonomous Targeting of Infectious Superspreaders Using Engineered Transmissible Therapies
Source: PLoS Comput Biol. 2011 Mar 17;7(3):e1002015. doi: 10.1371/journal.pcbi.1002015 (PMC3060167; doi:10.1371/journal.pcbi.1002015)
Supplement: Text S1 — Full description of the multi-scale model used to predict the effects of a TIP intervention on HIV-1 infection dynamics among a population of host individuals, within host individuals, and within host cells. This report contains detailed descriptions of each model, tables of parameters and state variables, supporting figures, an analysis of the sensitivity of the TIP model to changes in parameters, and an analysis of the sensitivity of the TIP model to changes in structure and changes in basic transmission biology. (PDF) [file pcbi.1002015.s001.pdf]

Supporting Information for:

Autonomous Targeting of Infectious Superspreaders using Engineered Transmissible Therapies

Vincent T. Metzger, James O. Lloyd-Smith and Leor S. Weinberger*

*To whom correspondence should be addressed. E-mail: lsweinberger@ucsd.edu

This file includes:

Methods

Models

Tables S1 to S7

Supporting Figures S1 – S3

Sensitivity Analysis

Supporting References

Abbreviations used:

SAC = Sexual Activity Class
TIP = Therapeutic Interfering Particle
ART = Antiretroviral Therapy
VL = Viral Load

Table of Contents

Introduction………………………………………………………………………….....4

Methods……………….……….…………….…………………….…………………...4

Models

Vaccine model (population level)……….….…………….…………...………..5

ART model (population level)…………………………………………...…12

TIP model (population level)......…..……….……………………………........…...15

TIP in the presence of ART model (population level)…………..………………22

Calculation of transmission-rate function…………………………….....……25

Calculation of the duration of the asymptomatic period……………………......27

Modeling TIP and HIV-1 at the individual-patient level (*in vivo* model)……29

Modeling TIP and HIV-1 at the single-cell level (intracellular model)..……..31

Mechanisms for increasing TIP abundance…………………………………..35

Considerations for TIP transmission to uninfected hosts…………………….37

Supporting Tables

S1 Population model parameters………………………………………………...40

S2 Population model state variables……………………………………………..42

S3 Population model transmission probability functions………………………..43

S4 *In vivo* model state variables…………………………………………………45

S5 Intracellular model state variables……………………………………………46

S6 *In vivo* model parameters……………………………………………………..47

S7 Intracellular model parameters………………………………………………48

Supporting Figures

Figure S1: rate of vaccine and ART rollout…………………………….………..49

Figure S2: Projected impact of TIP on AIDS prevalence and incidence...............50

Figure S3: Projected impact of non-transmitting TIPs…..………………..………..51

Sensitivity of TIP model to changes in parameters

Sensitivity of the TIP R_0 (i.e. ability of TIP to stably propagate)……………….53

Sensitivity to changes in risk structure………………………………………..58

Sensitivity to changes in population removal (death)………………………...59

Sensitivity to changes in AIDS patient death rate………………………………60

Sensitivity to changes in intracellular parameters……………………………….61

Sensitivity to changes in intracellular parameters (with BDI)…………………..62

Sensitivity of the TIP model to changes in structure and changes in basic transmission biology

Sensitivity of model when transmission function is re-scaled ………………...63

Sensitivity of model if TIP-infected individuals (S_T) revert to Susceptible individuals (S)…….………………………..………………………………..66

Sensitivity of model to removal of independent transmission of TIPs (i.e. removal of S_T individuals) ……………………………………….………………..71

Supporting References……………..……………….…...……………………………72

Introduction

In this supporting information section we describe the multi-scale model used to predict the effects of a TIP intervention on population level HIV-1/AIDS prevalence, as well as a model to predict the population-level effects of ART and of a hypothetical protective vaccine for HIV-1. The TIP model is composed of three constituent models describing HIV-1 infection dynamics at different hierarchical scales: (i) among a population of host individuals ("population level") (ii) within host individuals ("*in vivo*") (iii) within host cells ("intracellular"). Our multi-scale model specifies mechanistic links between each scale and the next scale of organizational complexity (intracellular → *in vivo* → population level). Transmission probabilities and the duration of the HIV-1 asymptomatic period are based on *in vivo* viral loads using relationships determined from epidemiological data. Viral loads under TIP intervention are predicted from the *in vivo* TIP model. The *in vivo* model in turn is based upon an intracellular model of retroviral packaging.

For familiarity, we begin by introducing the population-level models used to calculate the effect of a hypothetical protective vaccine for HIV-1 and for the effect of ART, respectively. The protective vaccine model and ART models utilize the same risk structure and many of the same functional forms used in the TIP population model.

Methods

All ODE models were coded in both Berkeley Madonna 8.3.9™ and Mathematica 7.0™ and numerical solutions were performed in both programs. Analytical solutions were obtained in Mathematica 7.0™.

Vaccine Model (Population Level)

The vaccine model is a simplified version of a risk structured model constructed from UNAIDS field-data collected from antenatal clinics in Malawi [1]. We refer to this model as the 'Baggaley model' and our simplified version includes the full risk-structure formulation with four distinct sexual activity classes (SACs). Since the goal of the model is a broad proof-of-concept analysis for TIP intervention, for simplicity we utilize a simplified formulation of 'disease age' where individuals progress from HIV-infected (asymptomatic) to AIDS in ~10 years. Also, this model does not assume the presence of anti-retroviral therapy. Individuals are classified by the following disease state: as susceptible (*S*), vaccinated (*V*), infected with HIV-1 (*I*) or as an AIDS patient (A_w). The subscript *w* denotes AIDS caused by wild-type HIV-1 infection. Supplemental Table S2 summarizes and describes these disease states. Individuals in the *S*, *V* and *I* disease-states are divided into SACs in accordance with field data (indicated by subscript *i*), while all individuals in the A_w class are assumed (as in [1]) to have sexual contacts at the rate corresponding to the lowest risk group (SAC 4) owing to their poor health. Hypothetical vaccination campaigns are characterized by the vaccine's protective effect f and by vaccine coverage p , and the model equations are as follows:

$$
\begin{aligned}
\frac{dS_i}{dt} &= \lambda\theta_i n_0(1-p) - \sigma(V_i,S_i)S_i - \beta_W^I C(S_i,I_i) - \beta_W^A C_A(S_i,A_w) - \mu S_i \\
\frac{dV_i}{dt} &= \lambda\theta_i n_0 p + \sigma(V_i,S_i)S_i - (1-f)\beta_W^I C(V_i,I_i) - (1-f)\beta_W^A C_A(V_i,A_w) - \mu V_i \\
\frac{dI_i}{dt} &= \beta_W^I C(S_i,I_i) + (1-f)\beta_W^I C(V_i,I_i) + (1-f)\beta_W^A C_A(V_i,A_w) + \beta_W^A C_A(S_i,A_w) - (\mu+\gamma_1)I_i \\
\frac{dA_w}{dt} &= \gamma_1 \sum_{i=1}^{4} I_i - (\mu+\alpha)A_w \\
\forall i &= 1,2,3,4
\end{aligned}
$$

Parameter definitions, values, and corresponding references are shown in Table S1. The contact function (*C*) as well as the function describing the scale-up of the vaccination campaign (σ) are not listed in Table S1 and are instead explained below. The

transmission probabilities per-partnership are denoted β_X^Y where Y represents the disease state of the source of the infection, and X represents the viral strain (which is wild-type HIV, denoted X=W, for the vaccine model). The per-partnership transmission probability β_W^I (describing transmission of wild-type HIV-1 by individuals in the *I* disease state) is set equal to 0.105 per partnership in agreement with the weighted average of [1], corresponding to a mean viral load of 10^5 copies/mL. β_W^A, the per-partnership probability of wild-type HIV-1 infection originating from an AIDS patient, is set to 0.54 per partnership which is the maximum transmission probability considered in the Baggaley model [1].

Contact between two individuals is represented by a contact function that considers asymmetric mixing of individuals among the four SACs:

$$C(X_i, Y) = c_i X_i \left[\varepsilon \left(\frac{Y_i}{N_i} \right) + (1-\varepsilon) \frac{\sum_{j=1}^{4} c_j Y_j}{\sum_{j=1}^{4} c_j N_j} \right]$$

This contact function describes an individual in disease state X becoming infected by an individual in disease state Y. The subscript *i* denotes the SAC, c_i is the average number of sexual partners per year in SAC *i*, and N_i is the sum of all sexually active individuals in SAC *i:*

$$N_i = S_i + I_i + V_i$$

Similarly, the subscript *j* denotes SAC *j*, c_j is the average number of sexual partners per year in SAC *j*, and N_j is the sum of all sexually active individuals in SAC *j.*

In this deterministic population model, all individuals are identical with the exception of their risk structure. Each individual belongs to one of the four SACs. Transmission probabilities and the duration of the asymptomatic phase of HIV infection are dictated by

steady-state viral loads as predicted by the within-host model. For example, all dually-infected individuals in SAC 1 have the same predicted transmission probability for a given TIP (as defined by the TIP's intracellular parameters).

In the contact function, ε is the degree of assortative mixing with $\varepsilon = 1$ corresponding to entirely assortative mixing and $\varepsilon = 0$ corresponding to entirely random mixing. The first term inside the brackets of the contact function describes assortative mixing in which infected individuals are encountered in proportion to their prevalence in SAC *i*. The second term describes random contacts in which infected individuals are encountered in proportion to their contribution to all of the sexual contacts being made in the entire population. We set the mixing parameter ε equal to 0.37, as estimated in [1].

Contacts in which AIDS patients (A_w) transmit wild-type virus are represented by a contact function modified to account for our assumption that all AIDS patients 'move' to SAC 4 as described by Baggaley et al. The contact function for AIDS patients is:

$$C_A(X_i, A_w) = \frac{c_i X_i c_4 A_w}{\sum_{i=1}^{4} c_i N_i}$$

The vaccination campaign is assumed to start after HIV/AIDS prevalence reaches steady-state in the Baggaley model without the vaccine (i.e. $V_i(0) = 0, p = 0, f = 0$). The function $\sigma(V_i, S_i)$ describes the scale-up of the vaccination campaign:

$$\sigma(V_i, S_i) = \max\left[0, \left(p - \frac{V_i}{V_i + S_i}\right) r\right]$$

The parameter *r* is a constant that controls the rate that susceptible individuals receive the vaccine immediately following the onset of the vaccination campaign. We set r = 2

years^{-1} for all four SACs. In the aggressive vaccination campaign described by $\sigma(V_i, S_i)$, we assume that initial rollout of the vaccination program will be exceptionally rapid such that the target coverage is reached in approximately 5 years (see Supplemental Figure S1). Initial conditions for V_i are calculated as 5% of the target coverage p multiplied by the initial conditions for each S_i (i.e. $V_i(0) = 0.05\, p\, [S_i(0)+ I_i(0)+A(0)]$ and $S_i(0)$, $I_i(0)$, and $A(0)$ are decremented appropriately to conserve total population size). Following vaccine introduction, a fraction p of incoming sexually active individuals are vaccinated as they first enter the sexually active population. Finally, we assume that vaccine protection is lifelong. We have deliberately made highly optimistic assumptions about the vaccine in order to compare the TIP to the best-case scenario for a protective vaccine.

Behavioral disinhibition is simulated as in [2] by increasing the contact rates c for all SACs.

HIV-1/AIDS prevalence is defined as:

$$\frac{\sum_i I_i + A_w}{\sum_i (S_i + I_i + V_i) + A_w}$$

AIDS prevalence is defined as:

$$\frac{A_w}{\sum_i (S_i + I_i + V_i) + A_w}$$

To calculate HIV-1 incidence per 100,000 individuals, we added 'reporter equations' that track the cumulative number of HIV-1 infections:

$$\frac{dI_{inc_i}}{dt} = \beta_W^I[C(S_i, I_i) + (1-f)C(V_i, I_i)] + \beta_W^A[(1-f)C_A(V_i, A_w) + C_A(S_i, A_w)]$$

We compute incidence per 100,000 individuals as:

$$\frac{I_{inc_i}(t) - I_{inc_i}(t-1)}{\sum_i [S_i(t) + I_i(t) + V_i(t)] + A_w(t)} \times 100{,}000$$

A similar 'reporter equation' is also used to compute AIDS incidence:

$$\frac{dA_{w_{inc}}}{dt} = \gamma_1 \sum_{i=1}^{4} I_i$$

and AIDS incidence per 100,000 individuals is defined as:

$$\frac{A_{w_{inc}}(t) - A_{w_{inc}}(t-1)}{\sum_i [S_i(t) + I_i(t) + V_i(t)] + A_w(t)} \times 100{,}000$$

Treatment performance of the vaccine is also evaluated by the predicted number of 'AIDS deaths averted' (in the total population, approximately 10^6 individuals). First, cumulative deaths from AIDS in the Baggaley Susceptible-Infected-AIDS model at time *t* are counted by the function D_{SIA}:

$$D_{SIA}(t) = \alpha(A_w(t))$$

Then, in the vaccine model, cumulative deaths from AIDS are counted by the function $D_{vaccine}$:

$$D_{vaccine}(t) = \alpha(A_w(t))$$

The number of AIDS deaths averted by the vaccination campaign is defined as:

AIDS deaths averted = (AIDS deaths during 100 years of epidemic without treatment) – (AIDS deaths during a 50 year epidemic followed by 50 years of treatment)

Or equivalently:

AIDS deaths averted = $D_{SIA}(t_{preVac} + t_{postVac}) - \left(D_{SIA}(t_{preVac}) + D_{vaccine}(t_{postVac})\right)$

t_{preVac} is defined as the length of time from the epidemic's initiation to the initiation of the vaccine campaign and $t_{postVac}$ is defined as the time elapsed after initiation of the vaccination campaign. In these simulations, t_{preVac} is 50 years and $t_{postVac}$ is also 50 years. This value of t_{preVac} is used because the model has reached an approximate steady-state by 50 years after the epidemic's initiation. Furthermore, we have employed information from the literature to estimate when a potential vaccine might optimistically be initiated in the context of the HIV/AIDS epidemic. Assuming that the HIV epidemic began in most African populations in ~1965 (with the initial zoonotic event likely occurring in the 1930s +/-20 years [3]) and the vaccination campaign was initiated in ~2015, then t_{preVac} = 50 years. Importantly, the results are qualitatively indistinguishable when t_{preVac} = 100 years, although larger values of t_{preVac} result in a higher number of total deaths attributed to the HIV/AIDS epidemic prior to the introduction of the hypothetical vaccination campaign.

The number of AIDS deaths with and without the vaccine is computed by comparing the number of AIDS deaths over a 100-year period in the absence of vaccine to the number of AIDS deaths during the 50 years after introduction of a vaccine (when the vaccine was introduced 50 years after the beginning of the epidemic).

The same basic mathematical framework as shown here to evaluate vaccine performance is also used to evaluate ART and TIP performance. Specifically, HIV/AIDS prevalence, AIDS prevalence, HIV-1 incidence, AIDS incidence, and ‘AIDS deaths averted’ are similarly computed for the ART population model, the TIP population model, and the TIP in the presence of ART population model.

ART Model (Population Level)

The ART model is also based upon our simplified Baggaley model [1]. As with the vaccine model, we have deliberately made optimistic assumptions about ART by, for example, assuming an optimistic 'Universal Test and Treat' scenario for ART [4]. Individuals are classified by disease-state as susceptible (S), infected with HIV-1 (I), receiving ART (I^{ART}), as an AIDS patient (A_w), or as an AIDS patient receiving ART (A_w^{ART}). ART is characterized by a coverage fraction p_{ARV}, ART attrition rate ϕ (i.e. the rate that patients fail therapy or are lost and has been extensively measured in sub-Saharan Africa [5,6,7]), ART efficacy ν, and ART 'rollout' parameter r_{art}, as summarized in Supplemental Table S1. The efficacy of modern ART in halting HIV-1 transmission is estimated to be 99% and is supported by [8]. The parameter t_0 is the time when ART is introduced and t is the time elapsed after ART introduction. For simplification, we do not consider drug-resistance and we assume that patients who experience ART failure move from the treated (ART) disease state back to the infected disease state and progress to AIDS at the same rate as drug-naïve patients (a very optimistic assumption for ART). The model equations are:

$$\frac{dS_i}{dt} = \lambda\theta_i n_0 - \beta_W^I C(S_i, I_i) - \beta_W^A C_A(S_i, A_w) - (1-\nu)\left[\beta_W^I C(S_i, I_i^{ART}) + \beta_W^A C_A(S_i, A_w)\right] - \mu S_i$$

$$\frac{dI_i}{dt} = \left[1 - p_{ART}(1 - e^{\frac{-(t-t_0)}{r_{art}}})\right]\left[\beta_W^I C(S_i, I_i) + \beta_W^A C_A(S_i, A_w) + (1-\nu)\begin{bmatrix}\beta_W^I C(S_i, I_i^{ART}) \\ + \beta_W^A C_A(S_i, A_w)\end{bmatrix}\right]$$
$$- \sigma\left(I_i, I_i^{ART}\right)I_i + \phi I_i^{ART} - (\mu + \gamma_1)I_i$$

$$\frac{dI_i^{ART}}{dt} = \left[p_{ART}(1 - e^{\frac{-(t-t_0)}{r_{ART}}})\right]\left[\beta_W^I C(S_i, I_i) + \beta_W^A C_A(S_i, A_w) + (1-\nu)\begin{bmatrix}\beta_W^I C(S_i, I_i^{ART}) \\ + \beta_W^A C_A(S_i, A_w)\end{bmatrix}\right]$$
$$+ \sigma\left(I_i, I_i^{ART}\right)I_i - (\mu + \phi)I_i^{ART}$$

$$\frac{dA_w}{dt} = \gamma_1 \sum_{i=1}^{4} I_i + \phi A_w^{ART} - \sigma\left(A_w, A_w^{ART}\right)A_w - (\mu + \alpha)A_w$$

$$\frac{dA_w^{ART}}{dt} = \sigma\left(A_w, A_w^{ART}\right)A_w - (\mu + \alpha + \phi)A_w$$

$\forall i = 1,2,3,4$

The rollout parameter r_{art} describes the rate that infected individuals receive ART immediately following the onset of the ART campaign. This rollout parameter results in approximately 75% coverage of new infections within 5 years. The function σ describes the rollout of ART:

$$\sigma(I_i, I_i^{ART}) = \max\left[0, \left(p_{ART} - \frac{I_i^{ART}}{I_i + I_i^{ART}}\right)r_{art}\right]$$

and

$$\sigma(A_w, A_w^{ART}) = \max\left[0, \left(p_{ART} - \frac{A_w^{ART}}{A_w + A_w^{ART}}\right)r_{art}\right]$$

We make the optimistic assumptions that (i) ART is administered in equal proportion (i.e. 75%) to each of the four SACs and (ii) the ART campaign is very aggressive such that the final coverage rate (i.e. 75% of all new infections receive ART) is reached within approximately 5 years (see Figure S1).

The mathematical methods used to evaluate vaccine performance (see section entitled 'Vaccine model (population level)') are also utilized to evaluate ART. HIV/AIDS prevalence is computed using the same method as (3) and is functionally similar to the HIV/AIDS prevalence calculation in the vaccine model. AIDS prevalence, HIV-1 incidence, AIDS incidence, and AIDS deaths averted are computed for this ART model using the previously described strategies implemented in the vaccine model.

TIP Model (Population Level)

The population TIP model is also based on the Baggaley model [1]. Individuals are classified as susceptible (S), HIV-1 infected (I), susceptible to HIV-1 but infected with TIP (S_T), dually infected with HIV-1 and TIP (I_d), as an AIDS patient with wild-type virus (A_w), or as a dually infected AIDS patient (A_d).

Contact between two individuals resulting in HIV-1 and/or TIP transmission is represented by the same contact function used in the above vaccine model, which accounts for asymmetric mixing of individuals between the four SACs.

Contacts between individuals in the TIP population model are weighted by statistically independent transmission probabilities (β) which are calculated from steady-state HIV-1 and TIP viral loads from the *in vivo* model (see section entitled 'calculation of transmission rate function'). There are six distinct transitions between infection classes in the TIP population model (see Supplemental Table S3 for details):

1) Conversion from S to I can occur by an S becoming infected with wild-type virus through contact with an I, I_d, A_w, or A_d at the following rates:

$$\beta_W^I C(S_i, I_i)$$

$$\beta_W^{I_d}(1-\beta_T^{I_d})C(S_i, I_{d_i})$$

$$\beta_W^A C_A(S_i, A_w)$$

$$\beta_W^{A_d}(1-\beta_T^{A_d})C_A(S_i, A_d)$$

Note that when S individuals contact dually-infected individuals (I_d or A_d) the TIP and wild-type HIV-1 are assumed to transmit independently. Thus the probability that an S individual converts to I due to contact with an I_d individual is

$\beta_W^{I_d}(1-\beta_T^{I_d})$ as shown above, while conversion to S_t and I_d occur with probabilities $\beta_T^{I_d}(1-\beta_W^{I_d})$ and $\beta_W^{I_d}\beta_T^{I_d}$, respectively (shown below).

2) The conversion of S to I_d occurs through transmission of both viruses from an I_d to an S or from an A_d to an S:

$$\beta_W^{I_d}\beta_T^{I_d}C(S_i, I_{d_i})$$

$$\beta_W^{A_d}\beta_T^{A_d}C_A(S_i, A_d)$$

3) Conversion of S to S_T occurs through contact of an S with an I_d or an S with an A_d in which TIP is transmitted and wild-type virus is not transmitted:

$$\beta_T^{I_d}(1-\beta_W^{I_d})C(S_i, I_{d_i})$$

$$\beta_T^{A_w}(1-\beta_W^{A_d})C_A(S_i, A_d)$$

4) S_T can be converted to I_d by becoming infected with the wild-type virus through contact with an I, I_d, A_w, or A_d:

$$\beta_W^{I}C(S_{t_i}, I_i)$$

$$\beta_W^{I_d}C(S_{t_i}, I_{d_i})$$

$$\beta_W^{A_w}C_A(S_{t_i}, A_w)$$

$$\beta_W^{A_d}C_A(S_{t_i}, A_d)$$

Note in this case that there is no consideration on whether TIP is transmitted (i.e. no dependence on $\beta_T^{I_d}$ or $\beta_T^{A_d}$) since TIP superinfection is not responsible for transitions between infection classes.

5) The conversion of I to I_d is catalyzed by contact of I with I_d or A_d in which TIP is transmitted:

$$\beta_T^{I_d} C(I_i, I_{d_i})$$

$$\beta_T^{A_d} C_A(I_i, A_d)$$

6) Finally, the conversion of A_w to A_d is catalyzed by transmission of TIP from either an I_d or A_d:

$$\beta_T^{A_d} C_A(A_w, A_d)$$

$$\beta_T^{I_d} C(A_w, I_{d_i})$$

The table below shows the per-partnership transmission probabilities for the statistically-independent transmission of either TIP or wild-type virions originating from dually-infected individuals. The representative TIPs in this table are characterized by their predicted reduction in viral load (VL).

**TIP**	$\beta_W^{I_d}$	$\beta_T^{I_d}$
0.5 Log VL Reduction	0.040	0.093
1.0 Log VL Reduction	0.013	0.107
1.5 Log VL Reduction	0.004	0.107

Note that the per-partnership transmission probability of $\beta_T^{I_d}$ approaches saturation for TIPs predicted to produce drastic reductions in set-point viral load. See section entitled 'Calculation of transmission-rate function' for details.

The Equations for the TIP population model are:

$$\frac{dS_i}{dt} = \lambda\theta_i n_0 - \beta_W^I C(S_i, I_i) - \beta_W^{I_d}(1-\beta_T^{I_d})C(S_i, I_{d_i}) - \beta_W^{I_d}\beta_T^{I_d}C(S_i, I_{d_i}) - \beta_T^{I_d}(1-\beta_W^{I_d})C(S_i, I_{d_i})$$
$$-\beta_W^A C_A(S_i, A_w) - \beta_W^{A_d}\beta_T^{A_d}C_A(S_i, A_d) - \beta_W^{A_d}(1-\beta_T^{A_d})C_A(S_i, A_d) - \beta_T^{A_d}(1-\beta_W^{A_d})C_A(S_i, A_d) - \mu S_i$$

$$\frac{dS_{t_i}}{dt} = \beta_T^{I_d}(1-\beta_W^{I_d})C(S_i, I_{d_i}) - \beta_W^I C(S_{t_i}, I_i) - \beta_W^{I_d}C(S_{t_i}, I_{d_i}) + \beta_T^{A_d}(1-\beta_W^{A_d})C_A(S_i, A_d)$$
$$-\beta_W^{A_d}C_A(S_{t_i}, A_d) - \beta_W^A C_A(S_{t_i}, A_w) - \mu S_{t_i}$$

$$\frac{dI_i}{dt} = \beta_W^I C(S_i, I_i) + \beta_W^{I_d}(1-\beta_T^{I_d})C(S_i, I_{d_i}) - \beta_T^{I_d}C(I_i, I_{d_i}) + \beta_W^A C_A(S_i, A_w) + \beta_W^{A_d}(1-\beta_T^{A_d})C_A(S_i, A_d)$$
$$-\beta_T^{A_d}C_A(I_i, A_d) - (\mu+\gamma_1)I_i$$

$$\frac{dI_{d_i}}{dt} = \beta_W^{I_d}\beta_T^{I_d}C(S_i, I_{d_i}) + \beta_W^I C(S_{t_i}, I_i) + \beta_W^{I_d}C(S_{t_i}, I_{d_i}) + \beta_T^{I_d}C(I_i, I_{d_i}) + \beta_T^{A_d}C_A(I_i, A_d)$$
$$+\beta_T^{A_d}\beta_W^{A_d}C_A(S_i, A_d) + \beta_W^{A_d}C_A(S_{t_i}, A_d) + \beta_W^A C_A(S_{t_i}, A_w) - (\mu+\gamma_2(V))I_{d_i}$$

$$\frac{dA_w}{dt} = \gamma_1\sum_{i=1}^{4} I_i - \beta_T^{I_d}C(A_w, I_{d_i}) - \beta_T^{A_d}C_A(A_w, A_d) - (\mu+\alpha)A_w$$

$$\frac{dA_d}{dt} = \gamma_2(V)\sum_{i=1}^{4} I_{d_i} + \beta_T^{I_d}C(A_w, I_{d_i}) + \beta_T^{A_d}C_A(A_w, A_d) - (\mu+\alpha)A_d$$

$$\forall i = 1,2,3,4$$

Simulation of this TIP population model is conducted as follows. The Baggaley model is allowed to reach steady-state and then a TIP is introduced to 1% of all individuals without any targeting to high-risk classes. Similar benefits were obtained using much more restrictive initial conditions (e.g. utilizing TIP as a therapy and targeting TIP to <1% of only I and A_W individuals in the least active SACs—SAC 3 and SAC 4—generates similar results to Fig 2 in the main text). The same parameter values used in the vaccine model are also used in the above TIP model; however, functions are used in place of parameters to describe quantities that depend on the specific design of the TIP such as: (i) transmission probabilities, and (ii) the duration of the asymptomatic period.

As described in further detail below, these quantities are calculated based on measured correlations between transmission, disease progression, and viral load [9,10] where viral load is predicted from the *in vivo* TIP model.

The parameters β_W^I and γ_1 are static parameters that represent the transmission probability and the duration of the asymptomatic phase of individuals infected with only wild-type virus. In contrast, the transmission probabilities in the presence of TIP and the duration of the asymptomatic phase in dually-infected individuals in the TIP population models are represented by functions of steady-state viral load (i.e. viral set point) as predicted by the *in vivo* model (see below). The function $\gamma_2(V)$ is used to compute the duration of the asymptomatic phase in dually-infected individuals, and is calculated on page 26 in the section below entitled 'Calculation of the Duration of the Asymptomatic Period'. Table S3 describes transmission-probability functions. Behavioral disinhibition is simulated as described for the vaccine model using the same method as Blower et al. [2].

Contacts in which AIDS patients with only wild-type virus (A_w) or dually-infected AIDS patients (A_d) transmit wild-type virus or TIP are represented by a function similar to the contact function previously presented in the vaccine model. This contact function is modified to account for all AIDS patients moving to SAC 4:

$$C_A(X_i, A_w) = \frac{c_i X_i c_4 A_w}{\sum_{i=1}^{4} c_i N_i}$$

And similarly,

$$C_A(X_i, A_d) = \frac{c_i X_i c_4 A_d}{\sum_{i=1}^{4} c_i N_i}$$

HIV-1/AIDS prevalence is defined as:

$$\frac{\sum_i I_i + A}{\sum_i \left(S_i + S_{t_i} + I_i + I_{d_i}\right) + A}$$

Where $A = A_w + A_\text{d}$

Importantly, the results reported in the main text are not dependent on using the above definition of HIV-1/AIDS prevalence. The effects of the vaccine and the TIP show similar trends to figure 2 (main text) when compared using AIDS disease prevalence (see figure S2) defined as:

$$\frac{A}{\sum_i \left(S_i + S_{t_i} + I_i + I_{d_i}\right) + A}$$

TIP performance is also evaluated by the predicted number of AIDS deaths averted (in the total population, approximately 10^6 individuals). As in the vaccine model, cumulative deaths from AIDS in the Susceptible-Infected-AIDS model at time t is defined as:

$$D_{SIA}(t) = \alpha(A_w(t))$$

In the TIP model, cumulative deaths from AIDS is defined as:

$$D_{TIP}(t) = \alpha(A_w(t) + A_d(t))$$

t_{preTIP} is defined as the time elapsed at steady-state of the Susceptible-Infected-AIDS model prior to TIP introduction and $t_{postTIP}$ is defined as the time elapsed after introduction of the TIP. Therefore, the number of AIDS deaths averted by the TIP is:

AIDS deaths averted = $D_{SIA}(t_{preTIP} + t_{postTIP}) - \left(D_{SIA}(t_{preTIP}) + D_{TIP}(t_{postTIP})\right)$

Modeling TIP in presence of ART (population level)

In this combined model, the TIP population model is modified to account for widespread ART. ART is implemented as described in the section entitled 'ART model (population level)'. As above, ART is characterized by the fraction of infected individuals treated (p_{ART}) and ART retention rate (ϕ) as summarized in Supplemental Table S1. Individuals are classified as susceptible (S), HIV-1 infected (I), Infected with HIV-1 and receiving ART (I^{ART}), susceptible to HIV-1 but infected with TIP (S_T), dually infected with HIV-1 and TIP (I_d), dually infected with HIV-1 and TIP while receiving ART (I_d^{ART}), as an AIDS patient with wild-type virus (A_w), as a dually infected AIDS patient (A_d), as a dually infected AIDS patient receiving ART (A_d^{ART}), or as an AIDS patient with wild-type virus receiving ART (A_w^{ART}). Steady-state values from the Baggaley model are used to compute initial conditions. Importantly, the rate that dually-infected individuals on ART fail to be retained in the treated population is assumed to be the same as for HIV-infected individuals. For clarity, it is worthwhile to point out one assumption made in the model: TIP transmission to individuals already infected with HIV-1 (I_i) is not targeted by the Universal-Test-and-Treat ART campaign because already infected individuals are assumed to have been missed during the testing (and treating) phase of the ART campaign. Essentially, this assumption states that if the ART campaign did not reach a set of individuals and they were infected by HIV, the same ART campaign will similarly be unlikely able to reach that set of individuals to halt TIP transmission. Thus, $C(I_i, I_{d_i})$ and $C_A(I_i, A_d)$ transmission terms will fall outside the ART coverage terms. The transmission equations are as follows:

$$
\begin{aligned}
\frac{dS_i}{dt} &= \lambda\theta_i n_0 - \beta_W^I C(S_i, I_i) - \beta_W^{I_d}(1-\beta_T^{I_d})C(S_i, I_{d_i}) - \beta_W^{I_d}\beta_T^{I_d}C(S_i, I_{d_i}) \\
&- \beta_T^{I_d}(1-\beta_W^{I_d})C(S_i, I_{d_i}) - \beta_W^A C_A(S_i, A_w) - \beta_W^{A_d}\beta_T^{A_d}C_A(S_i, A_d) \\
&- \beta_W^{A_d}(1-\beta_T^{A_d})C_A(S_i, A_d) - \beta_T^{A_d}(1-\beta_W^{A_d})C_A(S_i, A_d) \\
&+ (1-\nu)\left[\begin{array}{l}
- \beta_W^I C(S_i, I_i^{ART}) - \beta_W^{I_d}(1-\beta_T^{I_d})C(S_i, I_{d_i}^{ART}) \\
- \beta_T^{I_d}(1-\beta_W^{I_d})C(S_i, I_{d_i}^{ART}) - \beta_T^{I_d}\beta_W^{I_d}C(S_i, I_{d_i}^{ART}) \\
- \beta_W^A C_A(S_i, A_w^{ART}) - \beta_W^{A_d}\beta_T^{A_d}C_A(S_i, A_d^{ART}) \\
- \beta_W^{A_d}(1-\beta_T^{A_d})C_A(S_i, A_d^{ART}) - \beta_T^{A_d}(1-\beta_W^{A_d})C_A(S_i, A_d^{ART})
\end{array}\right] - \mu S_i
\end{aligned}
$$

$$
\begin{aligned}
\frac{dS_{t_i}}{dt} &= \beta_T^{I_d}(1-\beta_W^{I_d})C(S_i, I_{d_i}) - \beta_W^I C(S_{t_i}, I_i) - \beta_W^{I_d}C(S_{t_i}, I_{d_i}) \\
&+ \beta_T^{A_d}(1-\beta_W^{A_d})C_A(S_i, A_d) - \beta_W^{A_d}C_A(S_{t_i}, A_d) - \beta_W^A C_A(S_{t_i}, A_w) \\
&+ (1-\nu)\left[\begin{array}{l}
\beta_T^{I_d}(1-\beta_W^I)C(S_i, I_{d_i}) - \beta_W^I C(S_{t_i}, I_i^{ART}) - \beta_W^{I_d}C(S_{t_i}, I_{d_i}^{ART}) \\
+ \beta_T^{A_d}(1-\beta_W^{A_d})C_A(S_i, A_d^{ART}) - \beta_W^{A_d}C_A(S_{t_i}, A_d^{ART}) - \beta_W^A C_A(S_{t_i}, A_w^{ART})
\end{array}\right] - \mu S_{t_i}
\end{aligned}
$$

$$
\begin{aligned}
\frac{dI_i}{dt} &= \left(1 - p_{ART}\left(1 - e^{\frac{-(t-t_0)}{r_{ART}}}\right)\right)\left[\begin{array}{l}
\beta_W^I C(S_i, I_i) + \beta_W^{I_d}(1-\beta_T^{I_d})C(S_i, I_{d_i}) \\
+ \beta_W^A C_A(S_i, A_w) + \beta_W^{A_d}(1-\beta_T^{A_d})C_A(S_i, A_d)
\end{array}\right] \\
&+ (1-\nu)(1-p_{ART})\left[\begin{array}{l}
\beta_W^I C(S_i, I_i^{ART}) + \beta_W^{I_d}(1-\beta_T^{I_d})C(S_i, I_{d_i}^{ART}) \\
+ \beta_W^A C_A(S_i, A_w^{ART}) + \beta_W^{A_d}(1-\beta_T^{A_d})C_A(S_i, A_d^{ART}) \\
- \beta_T^{A_d}C_A(I_i, A_d^{ART})
\end{array}\right] \\
&- \beta_T^{A_d}C_A(I_i, A_d) - \beta_T^{I_d}C(I_i, I_{d_i}) - (\mu + \gamma_1)I_i + \phi I_i^{ART} - \sigma(I_i, I_i^{ART})I_i
\end{aligned}
$$

$$
\begin{aligned}
\frac{dI_i^{ART}}{dt} &= p_{ART}\left(1 - e^{\frac{-(t-t_0)}{r_{ART}}}\right)\left[\begin{array}{l}
\beta_W^I C(S_i, I_i) + \beta_W^{I_d}(1-\beta_T^{I_d})C(S_i, I_{d_i}) + \beta_W^A C_A(S_i, A_w) \\
+ \beta_W^{A_d}(1-\beta_T^{A_d})C_A(S_i, A_d)
\end{array}\right] \\
&+ (1-\nu)(p_{ART})\left[\begin{array}{l}
\beta_W^I C(S_i, I_i^{ART}) + \beta_W^{I_d}(1-\beta_T^{I_d})C(S_i, I_{d_i}^{ART}) \\
+ \beta_W^A C_A(S_i, A_w^{ART}) + \beta_W^{A_d}(1-\beta_T^{A_d})C_A(S_i, A_d^{ART})
\end{array}\right] \\
&- (\mu + \phi)I_i^{ART} - \sigma(I_i, I_i^{ART})I_i
\end{aligned}
$$

$$\frac{dI_{d_i}}{dt} = (1 - p_{ART}(1 - e^{\frac{-(t-t_0)}{r_{ART}}})) \begin{bmatrix} \beta_W^I C(S_{t_i}, I_i) + \beta_W^{I_d} \beta_T^{I_d} C(S_i, I_{d_i}) + \beta_W^{I_d} C(S_{t_i}, I_{d_i}) \\ + \beta_T^{A_d} C_A(I_i, A_d) + \beta_T^{A_d} \beta_W^{A_d} C_A(S_i, A_d) \\ + \beta_W^{A_d} C_A(S_{t_i}, A_d) + \beta_W^A C_A(S_{t_i}, A_w) \end{bmatrix}$$

$$+ (1-\nu)(1 - p_{ART}) \begin{bmatrix} \beta_W^I C(S_{t_i}, I_i^{ART}) + \beta_W^{I_d} \beta_T^{I_d} C(S_i, I_{d_i}^{ART}) + \beta_W^{I_d} C(S_{t_i}, I_d^{ART}) \\ + \beta_T^{A_d} C_A(I_i, A_d^{ART}) + \beta_T^{A_d} \beta_W^{A_d} C_A(S_i, A_d^{ART}) \\ + \beta_W^{A_d} C_A(S_{t_i}, A_d^{ART}) + \beta_W^A C_A(S_{t_i}, A_w^{ART}) \end{bmatrix}$$

$$+ \beta_T^{I_d} C(I_i, I_{d_i}) - (\mu + \gamma_2(V)) I_{d_i} + \phi I_{d_i}^{ART} - \sigma(I_{d_i}, I_{d_i}^{ART}) I_{d_i}$$

$$\frac{dI_{d_i}^{ART}}{dt} = p_{ART}(1 - e^{\frac{-(t-t_0)}{r_{ART}}}) \begin{bmatrix} \beta_W^I C(S_{t_i}, I_i) + \beta_W^{I_d} \beta_T^{I_d} C(S_i, I_{d_i}) + \beta_W^{I_d} C(S_{t_i}, I_{d_i}) \\ + \beta_T^{A_d} C_A(I_i, A_d) + \beta_T^{A_d} \beta_W^{A_d} C_A(S_i, A_d) \\ + \beta_W^{A_d} C_A(S_{t_i}, A_d) + \beta_W^A C_A(S_{t_i}, A_w) \end{bmatrix}$$

$$+ (1-\nu)(p_{ART}) \begin{bmatrix} \beta_W^I C(S_{t_i}, I_i^{ART}) + \beta_W^{I_d} \beta_T^{I_d} C(S_{t_i}, I_{d_i}^{ART}) + \beta_W^{I_d} C(S_{t_i}, I_{d_i}^{ART}) \\ + \beta_T^{A_d} C_A(I_i, A_d^{ART}) + \beta_T^{A_d} \beta_W^{A_d} C_A(S_i, A_d^{ART}) \\ + \beta_W^{A_d} C_A(S_{t_i}, A_d^{ART}) + \beta_W^A C_A(S_{t_i}, A_w^{ART}) \end{bmatrix}$$

$$+ \beta_T^{I_d} C(I_i, I_{d_i}) - (\mu + \phi) I_{d_i}^{ART} + \sigma(I_{d_i}, I_{d_i}^{ART}) I_{d_i}$$

$$\frac{dA_w}{dt} = \gamma_1 \sum_{i=1}^{4} I_i - \beta_T^{I_d} C(A_w, I_{d_i}) - \beta_T^{A_d} C_A(A_w, A_d) - (\mu + \alpha) A_w$$
$$- \sigma(A_w, A_w^{ART}) A_w + \phi A_w^{ART}$$

$$\frac{dA_d}{dt} = \gamma_2(V) \sum_{i=1}^{4} I_{d_i} + \beta_T^{I_d} C(A_w, I_{d_i}) + \beta_T^{A_d} C_A(A_w, A_d) - (\mu + \alpha) A_d$$
$$- \sigma(A_d, A_d^{ART}) A_d + \phi A_d^{ART}$$

$$\frac{dA_w^{ART}}{dt} = \sigma(A_w, A_w^{ART}) A_w - (\alpha + \mu + \phi) A_w^{ART}$$

$$\frac{dA_d^{ART}}{dt} = \sigma(A_d, A_d^{ART}) A_w - (\alpha + \mu + \phi) A_d^{ART}$$

$\forall i = 1,2,3,4$

Calculation of Transmission Rate Function

A substantial body of literature [9,10] has demonstrated that HIV-1 transmission rate is correlated with *in vivo* viral load. To calculate viral transmission we utilized a recent study by Fraser and colleagues [9] that analyzed the correlation between patient viral load and population-level HIV-1 transmission rate in a sub-Saharan African population. Fraser et al. found that transmission rate increases as HIV-1 viral load increases, but reaches a saturating point at high viral load that can be represented by the function:

$$\beta(V) = \frac{\beta_{\max} V}{V + \beta_{50}}$$

where β is the transmission probability per partnership, V is the viral load (expressed as copies/mL), and $\beta_{\max}$ and β_{50} are constants that set the maximum and half-saturation point of the transmission probability. In our model, we utilize this functional form with parameters selected to match key properties of the population on which the Baggaley model was based. Here we describe the parameterization of this curve used in our main analyses; we later consider the sensitivity of our results to an alternative parameterization (see section 'Sensitivity to re-scaling of the Fraser transmission function'). The maximum transmission probability per partnership $\beta_{\max}$ is set at 0.54, which corresponds to the highest transmission probability considered in the Baggaley model. To convert the transmission probability per partnership from the sub-Saharan African population analyzed in [9] to the sub-Saharan African population analyzed by [1], $\beta(V = 100{,}000)$ is set equal to 0.105 per partnership, which is the mean transmission probability from the Baggaley model. This means transmission probability corresponds to the mean viral load of 100,000 copies/mL (see Transmission Probability as a Function of Viral Load below). Because a TIP shares all the same viral coat proteins as HIV-1, the TIP transmission probability per partnership $\beta(V_T)$ is expressed as a function of identical form with the same parameter values as $\beta(V)$.

A plot of transmission probability per partnership as a function of viral load (copies/mL) shows saturation of transmission probability at high viral loads:

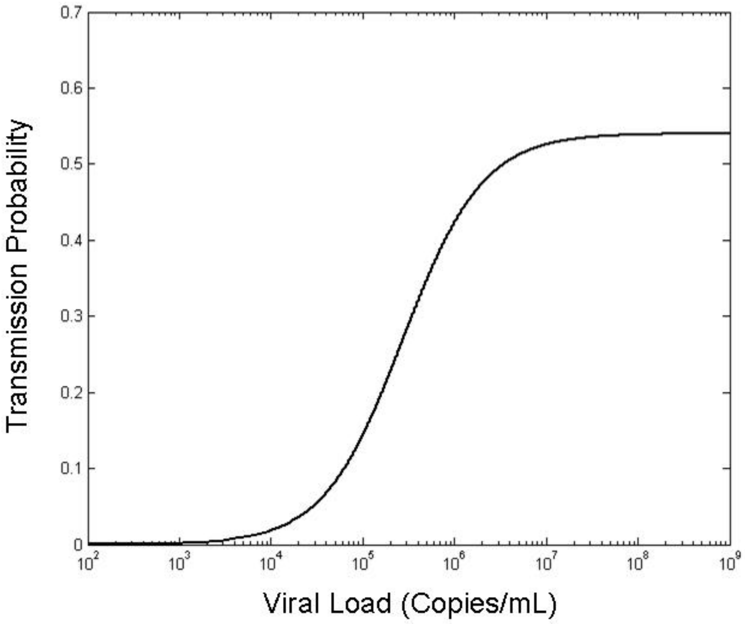

Predicted HIV-1 and TIP steady-state viral loads, calculated by analytically solving the *in vivo* model below, are implemented in the above transmission probability functions in order to compute the per-partnership transmission probabilities in the TIP population model.

A plot of HIV-1/AIDS prevalence as a function of single-cell TIP design parameters (main text, Figure 5c) reflects the dependence of the transmission probability function to changes in TIP parameters.

Calculation of the Duration of the Asymptomatic Period

To model the duration of the asymptomatic phase between infection and AIDS, we again borrow the empirically-derived formulation reported by Fraser et al [9]. The mean duration of the asymptomatic phase as a function of viral load is modeled with a decreasing Hill function [9]:

$$\gamma_2 = \gamma(V) = \left(\frac{\gamma_{\max} \gamma_{50}^{D_k}}{V^{D_k} + \gamma_{50}^{D_k}} \right)^{-1}$$

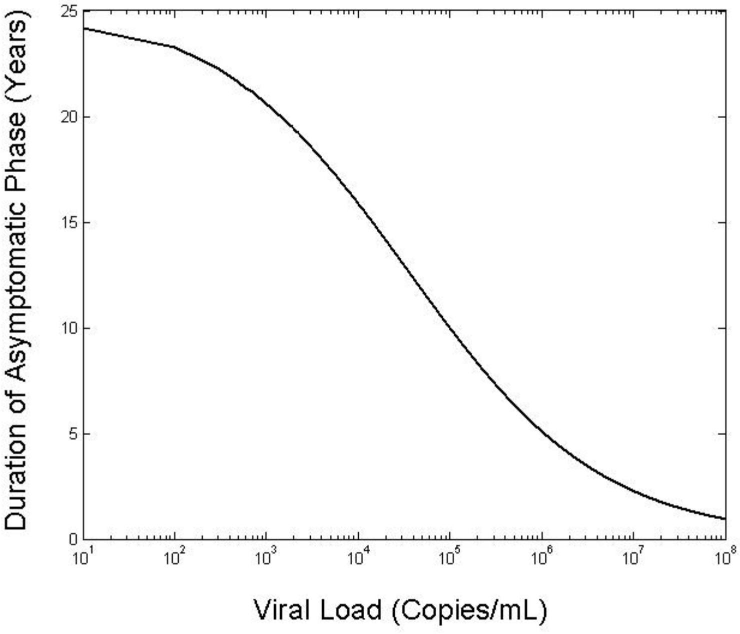

The disease progression rate is the inverse of the predicted duration of the asymptomatic period. The Hill coefficient of 0.41 and the maximum duration of the asymptomatic period (25.4 years) are adopted from [9], but γ_{50} is scaled so that a mean viral load of 100,000 copies/mL corresponds to an asymptomatic period of 10 years (as in (*1*)). This data-driven function is used to calculate the duration of the asymptomatic period based

on viral loads predicted from the *in vivo* TIP model (see section entitled 'Modeling TIP and HIV-1 infection at the individual-patient level').

Modeling TIP and HIV-1 infection at the individual patient level (*in vivo*)

The *in vivo* model of TIP and HIV-1 dynamics is based upon the established modeling framework for *in vivo* dynamics of HIV-1 [11,12] and we have previously described a similar model for the dynamics of a conditionally replicating virus *in vivo* [13]. Our model for dual infection is as follows:

$$\frac{dT}{dt} = b - dT - kVT - kV_T T$$
$$\frac{dI}{dt} = kVT - \delta I$$
$$\frac{dT_T}{dt} = kV_T T - dT_T - kVT_T$$
$$\frac{dI_D}{dt} = kVT_T - \xi\delta I_D$$
$$\frac{dV}{dt} = n\delta I - cV + \psi\xi n\delta I_D$$
$$\frac{dV_T}{dt} = \rho\psi\xi n\delta I_D - cV_T$$

The model tracks the dynamics of TIP virions (V_T) (indistinguishable from HIV-1 virions (V) except by RNA payload) within a host individual. Both viruses infect $CD4^+$ T cells (T) at the same rate k. HIV-1 infection converts T cells to productively infected cells (I), while TIP infection converts T cells to therapeutically infected cells (T_T) which do not produce any virus since a TIP only replicates conditionally in the presence of HIV-1. T_T cells are functionally equivalent to T cells. When HIV-1 infects T_T cells, the resulting dually infected cells produce both HIV-1 and TIP. In I_D cells, TIPs can downregulate HIV-1 production via antiviral payload genes and also compete for packaging with HIV-1 mRNA transcripts through increased abundance of cytoplasmic TIP mRNA.

Two intracellular parameters, P and D, describe properties of a TIP at the intracellular scale (see section entitled 'Modeling TIP and HIV-1 infection at the single-cell level'). The parameter P is the fold increase in TIP mRNA concentration as compared to HIV-1

mRNA in the cytoplasm of dually-infected cells. The parameter D is the fractional downregulation or upregulation of HIV-1 virion production by dually- infected cells. These intracellular parameters P and D are represented in this *in vivo* model by three functions (ρ, ψ, and ξ) which are obtained via steady-state analysis of the Intracellular Model (See section entitled 'Modeling TIP and HIV-1 at the single-cell level'). The function ρ represents the effective upregulation of TIP relative to HIV-1, the function ψ accounts for the downregulation of HIV-1 in a dually infected cell compared to a cell that is only infected with HIV-1, and the function ξ reflects the increased lifetime of a dually infected cell compared to a cell that is only infected with HIV-1 (note that $\xi \leq 1$ so the per capita death rate of dually infected cells is reduced). Parameters are adopted from a previously described *in vivo* model of dual infection [13] and are summarized in Supplemental Table S6. State variables in the *in vivo* model are explained in Supplemental Table S4.

Steady-state HIV-1 (V^*) and steady-state TIP (V_T^*) viral loads are used to calculate HIV-1 and TIP transmission within the population model based upon measured rates of HIV-1 transmission within a sub-saharan African population [9]. (see section entitled 'Calculation of transmission rate function'). The steady-state HIV-1 viral load is also used to calculate the duration of the asymptomatic period following infection (see section entitled 'Calculation of the duration of the asymptomatic period').

The previously reported model [13] differed slightly from the current model in that it considered engineered packaging signals in a conditionally replicating "cr-HIV-1" gene therapy vector that did not necessarily dimerize with wild-type HIV-1 packaging signal. The model presented here assumes that the TIP uses wild-type HIV-1 packaging signals, such that dimerization between TIP and wild-type HIV-1 packaging signals can occur as detailed explicitly in the next section.

Modeling TIP and HIV-1 infection at the single-cell level (intracellular model)

We introduce a simple model of intracellular dynamics to consider the key mechanisms underlying the production and packaging of TIP and HIV-1 retroviruses by dually infected cells. Our model tracks the production of genomic mRNA and its packaging into virions by assuming, based on experimental data [14], that all other packaging materials are present in excess compared to genomic mRNA and that genomic mRNA is the limiting reagent for packaging. The intracellular model of HIV-1 compares packaging of viral genomic mRNA $\left(G_W^S\right)$ into diploid genomic mRNA within virions $\left(G_{WW}^S\right)$. The superscript 'S' denotes cells singly infected with HIV-1 and the superscript 'Δ' denotes cells dually infected with HIV-1 and TIP. In the absence of a TIP, HIV-1 genomic mRNA is transcribed at a rate θ and packaged at a rate k_{pkg}. The rate constant k_{pkg} accounts for the dimerization of two genomic mRNAs. The model assumes that mRNA dimerization is a requirement for packaging and is the rate-limiting step, based on recent experimental data [14]. The model for HIV-1 intracellular dynamics in the absence of TIP is thus:

$$\frac{dG_W^S}{dt} = \theta - 2k_{pkg} \cdot {G_W^S}^2$$
$$\frac{dG_{WW}^S}{dt} = k_{pkg} \cdot {G_W^S}^2$$

In a dually infected cell where TIP is present, there are two species of genomic mRNA within the cell: wild-type HIV-1 genomic mRNA $\left(G_W^\Delta\right)$ and TIP genomic mRNA $\left(G_T^\Delta\right)$. There are three classes of possible diploid genomes: homozygous wild-type $\left(G_{WW}^\Delta\right)$, homozygous TIP $\left(G_{TT}^\Delta\right)$, and heterozygous diploid genomes $\left(G_{TW}^\Delta\right)$. See Supplemental Table S5 for descriptions of state variables in the Intracellular model. The TIP mRNA is produced at a greater rate P and could encode a downregulation factor that inhibits transcription of both mRNAs at a rate D similar to the previously described model [13].

As previously described [13], HIV-1 gene products are produced in proportion to HIV-1 mRNA and are largely responsible for HIV-1 cytotoxicity of infected cells. Therefore, we introduce ξ which is the factor by which the death rate of dually-infected cells is reduced compared to cells only infected with HIV-1. ξ is defined as:

$$\xi = 1 - D$$

However, the functional form of ξ does not appear in the steady-state solutions of *V* or V_t in the *in vivo* model and thus we relax the $\xi = 1 - D$ assumption and instead make the assumption of $\xi = 1$ (that dually infected cells do not have an increased lifetime compared to HIV-1 infected cells). This assumption (or any other assumption for the functional form of ξ) does not alter the *in vivo* or corresponding population level results.

The model tracks the production of wild-type HIV-1 genomic mRNA and TIP genomic mRNA and the packaging of these genomes into the three types of diploid genomes:

$$\frac{dG_W^\Delta}{dt} = (1-D)\cdot\theta - 2k_{pkg}\cdot {G_W^\Delta}^2 - k_{pkg}\cdot G_W^\Delta \cdot G_T^\Delta$$

$$\frac{dG_T^\Delta}{dt} = P\cdot(1-D)\cdot\theta - 2k_{pkg}\cdot {G_T^\Delta}^2 - k_{pkg}\cdot G_W^\Delta \cdot G_T^\Delta$$

$$\frac{dG_{WW}^\Delta}{dt} = k_{pkg}\cdot {G_W^\Delta}^2$$

$$\frac{dG_{TW}^\Delta}{dt} = 2k_{pkg}\cdot G_W^\Delta \cdot G_T^\Delta$$

$$\frac{dG_{TT}^\Delta}{dt} = k_{pkg}\cdot {G_T^\Delta}^2$$

Importantly, this intracellular model accounts for heterozygous virion production (G_{TW}^Δ) occurring in essentially a binomial partitioning, as recently reported [14].

By analytically solving for the steady states of this model, we relate these equations to the *in vivo* model parameters P and D by the following argument. We assume that the single stranded genome abundances G_W^Δ and G_T^Δ reach their steady states $\overline{G_W^\Delta}$ and $\overline{G_T^\Delta}$ quickly, so the rates of increase of G_{WW}^Δ and G_{TT}^Δ become constant values. Steady-state G_W^Δ and G_T^Δ are given by:

$$\frac{dG_W^\Delta}{dt} = 0 = (1-D)\cdot\theta - 2k_{pkg}\cdot {G_W^\Delta}^2 - k_{pkg}\cdot G_W^\Delta\cdot G_T^\Delta$$
$$\frac{dG_T^\Delta}{dt} = 0 = P\cdot(1-D)\cdot\theta - 2k_{pkg}\cdot {G_T^\Delta}^2 - k_{pkg}\cdot G_W^\Delta\cdot G_T^\Delta$$

Upon simplification:

$$\overline{{G_W^\Delta}^2} = \frac{(1-D)\cdot\theta}{2k_{pkg}(1+P)}$$
$$\overline{{G_T^\Delta}^2} = \frac{P^2(1-D)\cdot\theta}{2k_{pkg}(1+P)}$$

Because the packaging materials are assumed to be present in excess, the total quantities of different virion types produced by a cell before it dies are proportional to the rates of virion production. The relative quantities are thus determined by ratios of these rates. ρ , the effective upregulation of TIP compared to HIV-1, is determined to be equal to P^2 by the following calculation:

$$\rho = \frac{dG_{TT}^\Delta / dt}{dG_{WW}^\Delta / dt} = \frac{2k_{pkg}\left(\overline{G_T^\Delta}\right)^2}{2k_{pkg}\left(\overline{G_W^\Delta}\right)^2} = \frac{\left(\frac{(1-D)\theta}{2k_{pkg}}\times\frac{P^2}{(1+P)}\right)}{\left(\frac{(1-D)\theta}{2k_{pkg}}\times\frac{1}{(1+P)}\right)} = P^2$$

ψ is the downregulation of HIV-1 in a dually-infected cell compared to a cell only infected with wild-type HIV-1 and is determined to be equal to $\frac{1-D}{1+P}$ as follows. With G_{WW}^{S} as the abundance of homozygous wild-type HIV-1 genomes in a cell singly infected with HIV-1 and G_{WW}^{Δ} as the abundance of homozygous wild-type HIV-1 genomes in a cell dually-infected with both HIV-1 and TIP. ψ is therefore defined as:

$$\psi = \frac{dG_{WW}^{\Delta}/dt}{dG_{WW}^{S}/dt} = \frac{\left(\frac{(1-D)\theta/2k_{pkg}}{1+P}\right)}{\theta/2k_{pkg}} = \frac{1-D}{1+P}$$

Note that in general the lifespans of singly and dually infected cells may differ by a factor of ξ, which would appear in the denominator of this expression. If dually infected cells live longer, the ratio of HIV-1 virions to TIP virions produced by a dually infected cell would not be altered. Based on the transcription rate (θ=1,000 transcripts per hour) and packaging rate constant ($k_{pkg=}$100 transcripts^{-1} hour^{-1}) from the literature (see Supplemental Table S7), G_{W}^{S}, G_{W}^{Δ}, and G_{T}^{Δ} all reach steady-state very rapidly (within minutes). Representative trajectories are displayed below:

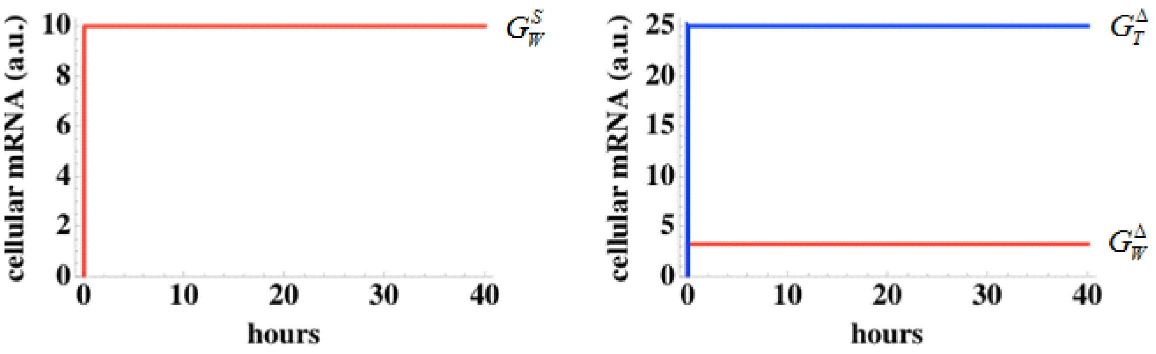

Left: HIV-1 genomic RNA G_{W}^{S} in single-infected cell (i.e. no TIP). Right: HIV-1 and TIP genomic RNA in dually-infected cell, G_{W}^{Δ} in **red**, G_{T}^{Δ} in **blue**).

Mechanisms for increasing TIP abundance

The overexpression of the TIP genome relative to HIV-1 arises from two molecular mechanisms. The first mechanism parallels the classical, well-established mechanism for over-expression of an interfering particle genome relative to a wild-type virus: the shorter genomic length of the interfering particle allows the interfering particle to transcribe more full-length genomes per unit time. This mechanism has been extensively described for RNA viruses such as VSV (for review see: [15]). The full-length HIV-1 genome is ~9.5kb genome while the TIP genome encoding only the cis-acting factors could be as small as ~3kb. HIV-1 would be unlikely to compete by acquiring a shorter genome as it would require sacrificing essential genes. A second mechanism for generating over-expression of TIP genomic RNA relative to HIV-1 genomic RNA is more specific to retroviruses and lentiviruses. This second mechanism involves the reengineering of splice-donor and splice-acceptor sites to ablate splicing such that TIP expresses only a single genomic RNA species that competes with HIV-1 genomic RNA more effectively due to increased abundance. Since wild-type HIV-1 can generate over a dozen different subgenomic mRNA splice variants that compete for packaging into virions [16], re-engineering of splice sites would increase the relative abundance of TIP genomic RNA relative to HIV-1 genomic RNA. This has been demonstrated experimentally, as mutation of the SIV and HIV-1 splice donor and acceptor sites has been shown to increase the abundance of unspliced mRNA relative to spliced HIV-1 and SIV mRNA [17,18]. To our knowledge, the most direct example of using splice-site mutation to increase full-length lentiviral genomic RNA and increase the titer of lentiviral production is a recent paper by Koldej and Anson [19] who show that mutations in the major and minor splice donors lead to reduced splicing, increased genomic RNA, and ultimately increased viral titer in HIV-based gene-therapy vectors. Bohne et al. [20] also specifically tested the hypothesis that mutating HIV-1 splice donors ablates splicing and allows for creation of more genomic RNA compared to wt-HIV-1 and demonstrated that this effect could be achieved. As with a shorter genome, it is unlikely that HIV-1 could acquire this phenotype because HIV-1 requires splicing to express its envelope gene *Env* and its accessory genes *Vif, Vpu, Vpr, Tat, Rev,* and *Nef*, so an HIV-1 mutant with a

splicing mutation could not replicate alone [21] and would require a helper virus (i.e. it would become a *conditionally* replicating virus just like a TIP).

Considerations for TIP transmission to uninfected hosts

In this section, we review the biology and physiology of viral transmission to examine whether TIPs could transmit in the absence of HIV. First, we consider the evidence that a single 'founder' virion or infected cell establishes HIV infection ~70% of the time [22]. If only a single virion initiates infection the transmission potential of TIP would be reduced. However, there is evidence from other studies that multiple viruses are transmitted in high-risk MSM populations and that the virus undergoes a genetic 'bottleneck' and subsequent selection for the most-fit variant [23]. Even in studies reporting that infection is initiated by a single virus, there is strong evidence that in high-risk individuals HIV infection is initiated by multiple strains of virus [22,24,25] arguing that the superspreaders are getting the most viruses, and hence are most likely to get TIP infections too. A related point that arises is whether a TIP that is transferred along with HIV will undergo a genetic bottleneck (such as the bottleneck imposed upon two competing HIV variants). As Haaland et al (2009) point out: "*studies in rhesus macaques in which relatively large doses of a virus quasispecies have been applied to hormonally thinned vaginal mucosa showed that multiple viruses initiate a localized infection, but that only a subset is able to establish a systemic infection [26,27]. In the current study, we cannot differentiate between an "outgrowth" model in which multiple variants initially establish a localized infection and only a single variant can generate a systemic infection, and a "mucosal barrier" model in which the genetic bottleneck is the result of a single HIV-1 variant with the capacity to penetrate the mucosal barrier."* These studies suggest that multiple variants may be able to initiate foci of infection within the genital compartment, but only a fraction of these can extend the infection

beyond the mucosa. This localized versus systemic point is highly relevant to TIP transmission since localized infection is sufficient for TIPs, which only need to infect one target cell and do not undergo the same competitive bottleneck as two HIV strains; thus additional TIP virions will actually have a higher probability of transmission than additional competing HIV strains. Importantly, our statistical treatment of transmission (see section entitled 'Calculation of transmission rate function') is consistent with both 'founder effects' and 'bottlenecking'.

Second, we consider the fact that without HIV there is no TIP replication in the host and to have any effect, (i) a single (or, at most, a few) TIP-infected cell must survive until subsequent challenge with HIV, and (ii) the TIP-infected cell must then be infected by HIV to initiate TIP replication in the host. The TIP (being a lentiviral vector) will integrate into the cell's genome and will remain in this cell for the lifetime of the cell. These lentivirally-transduced cells have the potential to be maintained for the lifetime of the individual, and gene-therapy trials show that they are definitely maintained for over a decade [28]. Most relevant is evidence from gene therapy transfer of autologous lentivirally-transduced CD4+ T cells, which indicates that these cells can be maintained in the individual for at least five years at this point in time [29].

Another crucial observation is that during the initial 'acute' stage of infection when viremia peaks within the patient, >50% of CD4+ T cells become infected [30]. Thus, the TIP-infected cell is likely to be infected by HIV at some point during initial/acute infection. In fact, the Phase I clinical trial by Carl June's group demonstrates strong

evidence that even in HIV-infected patients where the percentage of HIV-infected cells is typically ~1%, HIV can find and infect a relatively small percentage of lentivirus-transduced CD4+ T cells and mobilize the lentiviral vectors from these cells [29]. This last point that HIV encounters and superinfects previously infected cells is unsurprising given evidence from rhesus macaque SIV studies that dually-infected cells are common and that recombination occurs at an appreciable rate within animals, despite the low <1% frequency of HIV-infected CD4+ T cells [31,32]. These studies performed co-infection of rhesus macaques with *vpx*-deleted and *nef*-deleted SIV strains. Within days full-length recombinant +vpx+nef SIV could be isolated from the infected macaques. The recombinant +vpx+nef virus could only result from viral recombination within dually-infected cells. The fact that this recombination happens very quickly after initial infection indicates that dual infection of cells is appreciable and is happening early, not just during late-stage syncytia formation. Thus, we feel there is experimental precedent that: (i) TIP-infected cells could survive for months to years in the absence of HIV-infection, and (ii) despite TIP-infected cells being present at a very low frequency, HIV is likely to infect these cells during initial infection or subsequent infection.

In summary, we feel there is physiological rationale for arguing that TIPs could transmit in the absence of HIV. However, even if TIPs cannot transmit without HIV, the projected impact of TIP intervention is still substantial (see section entitled 'Sensitivity of model to removal of independent transmission of TIPs (i.e. removal of S_T individuals)') and appears qualitatively the same as Figure 2 (main text).

Table S1 – Parameters used in Population Models

Symbol	Values	Description	Sources
ε	0.37	Mixing pattern where 1 is completely assortative pairing and 0 is completely random pairing.	[1]
c_i	152.8, 13.6, 0.5, 0.2 partners per year for *i*=1,2,3,4 respectively	Annual partner change rate for individuals in SAC *i*	[1]
θ_i	0.1%, 26%, 59%, 15% for *i*=1,2,3,4 respectively	Fraction of new sexually active individuals recruited into SAC *i*	[1]
n_0	1,000,000	Initial population size (at *t*=0)	[1]
λ	0.03	Entry rate into the Susceptible population	[1]
μ	1/35 years = 0.029 years^{-1}	Rate at which individuals leave the model population	Corresponds to adults aged 15-49 years
α	1/2.5 years = 0.4 years^{-1}	Rate at which AIDS patients die from AIDS	[33]
γ_1	1/10 years = 0.1 years^{-1}	Rate at which HIV-1 infected individuals progress to AIDS	[34]
p	Range = 0.0 to 1.0 (used 0.8 and 0.95)	Vaccine coverage level (proportion of entering susceptibles who receive vaccine)	[34,35,36]
f	Range = 0.0 to 1.0 (used 0.3 and 0.5)	Vaccine efficacy	[34,36,37]
β_W^A	0.54 per partnership	Probability of wild-type HIV-1 transmission originating from an AIDS patient (This transmission probability value represents transmission of wild-type virus in the absence of a TIP and is a static parameter)	[1]
β_W^I	0.105 per partnership	Probability of wild-type HIV-1 transmission by an individual	[1]

		infected only with HIV-1. (This transmission probability value represents transmission of wild-type virus in the absence of a TIP and is a static parameter)	
r	2 years^{-1}	Rollout rate for initiation of vaccination campaign	Optimistic assumption (yields target coverage in ~5 years see Figure S1)
r_{ART}	0.345 years^{-1}	Rollout rate for initiation of ART treatment campaign	Optimistic assumption (yields target coverage in ~5 years)
p_{ART}	75%	Fraction of new infections treated by ART	"optimistic" (50% ART Coverage is goal in South Africa.) [5]
ν	99%	Efficacy of Modern HAART in halting HIV-1 transmission	[4]
ϕ	0.5/year	ART failure/attrition rate	[5,6,7]

Table S2 – State Variables used in Population Models

State Variable	Description
S_i	Susceptible individuals in SAC *i*
I_i	Individuals infected with HIV-1 in SAC *i*
V_i	Vaccinated individuals in SAC *i*
S_{t_i}	Individuals infected with TIP in SAC *i*
I_{d_i}	Individuals dually infected with both HIV-1 and TIP in SAC *i*
I_i^{ART}	ART-treated individuals infected with HIV-1 in SAC *i*
$I_{d_i}^{ART}$	ART-treated individuals dually infected with both HIV-1 and TIP in SAC *i*
A_w	AIDS patients infected with wild-type virus
A_d	AIDS patients dually infected with HIV-1 and TIP
A_w^{ART}	AIDS patients infected with wild-type virus receiving ART
A_d^{ART}	AIDS patients dually infected with HIV-1 and TIP receiving ART

Table S3 – Transmission Probabilities and Transmission Probability Functions

Symbol	Description of Transmission Probability	Values	Rationale
β_W^I	Transmission of wild-type HIV-1 by an individual infected only with HIV-1 (static parameter independent of TIP)	0.105 per partnership	[1]
$\beta_W^{I_d}$	Wild-type HIV-1 transmission originating from a dually infected individual (function of TIP design parameters)	0.013 per partnership for a TIP with 1.0 Log VL reduction	Statistically Independent Transmission [9]
$\beta_T^{I_d}$	TIP transmission originating from a dually infected individual (function of TIP design parameters)	0.107 per partnership for a TIP with 1.0 Log VL reduction	Statistically Independent Transmission [9]
$\beta_W^{A_d}$	Wild-type HIV-1 transmission originating from an AIDS patient also infected with TIP (function of TIP design parameters)	0.013 per partnership for a TIP with 1.0 Log VL reduction	Statistically Independent Transmission [9]
$\beta_T^{A_d}$	TIP transmission originating from a dually infected AIDS patient (function of TIP design parameters)	0.107 per partnership for a TIP with a 1.0 Log VL reduction	Statistically Independent Transmission [9]
β_W^A	Wild-type HIV-1 transmission originating from an AIDS patient (static	0.54 per partnership	[1]

	parameter independent of TIP)		
$\beta_T^{S_t}$	TIP transmission originating from an individual only infected with therapy virus.	0 per partnership	TIPs replicate only in the presence of wild-type virus

<u>Table S4 – State Variables for the *in vivo* model</u>

State Variable	Description
T	$CD4^+$ T lymphocytes
I	Cells infected with HIV-1
V	HIV-1 Viral Load
I_T	Therapeutically infected cells
I_D	Cells infected with both HIV-1 and TIP
V_T	TIP Viral Load

Table S5 – State Variables for the Intracellular Model

State Variable	Description
G_W^S	Wild-type genomic mRNA in a cell that is singly-infected with HIV-1
G_{WW}^S	Homozygous dimers of wild-type mRNA genomes in a cell that is singly-infected with HIV-1
G_W^Δ	Wild-type genomic mRNA in a cell that is dually-infected with HIV-1 and TIP
G_T^Δ	TIP genomic mRNA in a cell that is dually-infected with HIV-1 and TIP
G_{WW}^Δ	Homozygous dimers of wild-type mRNA genomes in a cell that is dually-infected with HIV-1 and TIP
G_{TT}^Δ	Homozygous dimers of TIP mRNA genomes in a cell that is dually-infected with HIV-1 and TIP
G_{TW}^Δ	Heterozygous dimers of TIP and wild-type mRNA genomes in a cell that is dually-infected with HIV-1 and TIP

Table S6 – Parameters used in Individual Patient TIP Model

**Parameter**	**Description**	**Value [*units*]**	**Sources**
b	Birth rate constant of uninfected $CD4^+$ T cells (T)	31 [*cells/(μL×day)*]	[38]
d	Death rate of uninfected CD4 T cells (T)	0.02 [*1/day*]	[39]
k	Infection rate of activated CD4 T cells per virion	1.875×10^{-4} [*μL/virions*]	No Data
δ	Death rate of HIV-1 infected cells (I)	0.7 [*1/day*]	[40,41]
n	Burst size; # of virions released from HIV-1 infected cell (I)	200 [dimensionless]	[42]
c	Clearance rate of HIV-1 (V) and TIP (V_T) virions	30 [*1/day*]	[40,43]
P	Fold increase in TIP mRNA concentration as compared to HIV-1 mRNA in cytoplasm of dually infected cells (I_D)	Varied from 1 to 35 [dimensionless]	††
D	Fractional downregulation ($0 < D < 1$) or upregulation ($D < 0$) of HIV-1 virion production from I_D cells	Varied from -1 to 1 [dimensionless]	††
T_0	Initial concentration of uninfected $CD4^+$ T cells in plasma	800 [*cells/μL*]	[11]
V_0	Initial HIV-1 set-point (before TIP treatment)	1×10^5 [*virions/mL*]	[11]
ξ	The factor by which the death rate of dually-infected cells is reduced compared to cells only infected with HIV-1	$\xi = 1 - D$, assumption used in simulations: $\xi = 1$	*
ρ	The effective upregulation of TIP compared to HIV-1	$\rho = P^2$	Derived
ψ	The downregulation of HIV-1 in a dually-infected cell compared to a cell only infected with wild-type HIV-1	$\psi = \frac{1-D}{1+P}$	Derived

†† = The TIP can be engineered to change the value of this parameter (for 0.5 Log TIP: {$P = 5.5$, $D = 0$}; for 1.0 Log TIP {$P = 13.3$, $D = 0$}; for 1.5 Log TIP {$P = 35$, $D = 0$})
* = Functional form does not affect steady-state

Table S7 – Table of Parameters used in Intracellular TIP Model

Parameter	Description	Value	Sources
θ	Transcription rate	1000 transcripts / hour	[44,45,46,47]
k_{pkg}	Packaging rate constant	100 transcripts^{-1} hour^{-1}	[48]

<u>Supporting Figure S1: Vaccine and ART rollout</u>

Top panel shows the rollout of the aggressive vaccination campaign in terms of the fraction of susceptible individuals that have been vaccination versus time. **Bottom panel** shows the rollout of the aggressive universal 'test-and-treat' ART campaign in term of fraction of infections that are ART treated over time. Both approaches reach their target coverage within approximately 5 years.

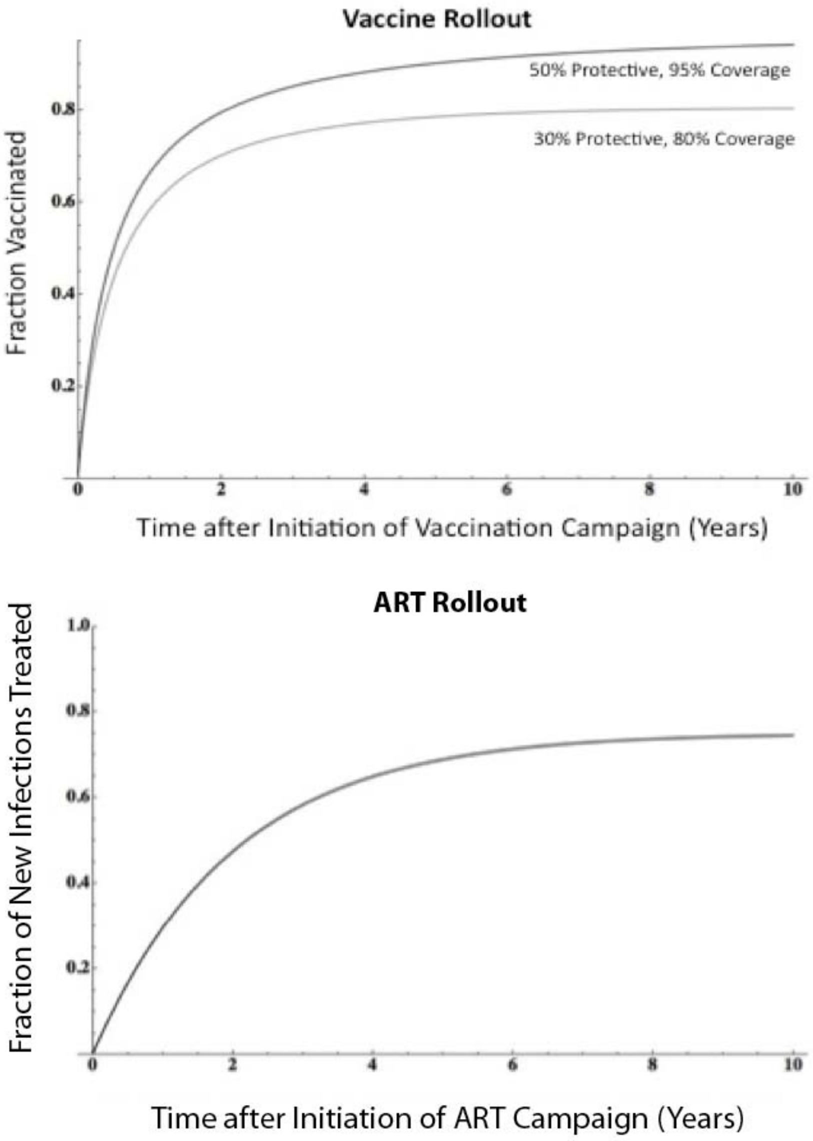

__Supporting Figure S2: Projected impact of a TIP on AIDS prevalence and incidence__

Panel A shows trajectories of 'Fraction living with AIDS' based on several intervention options including two partially protective vaccines, the highly optimistic treatment of 75% of infected patients with ART, and a shaded region representing the predicted impact of TIP interventions ranging from a TIP that produces a 0.5 Log viral load reduction to a TIP capable of achieving a 1.5 Log viral load reduction. **Panel B** shows the same simulations compared via AIDS incidence-per-100,000 individuals.

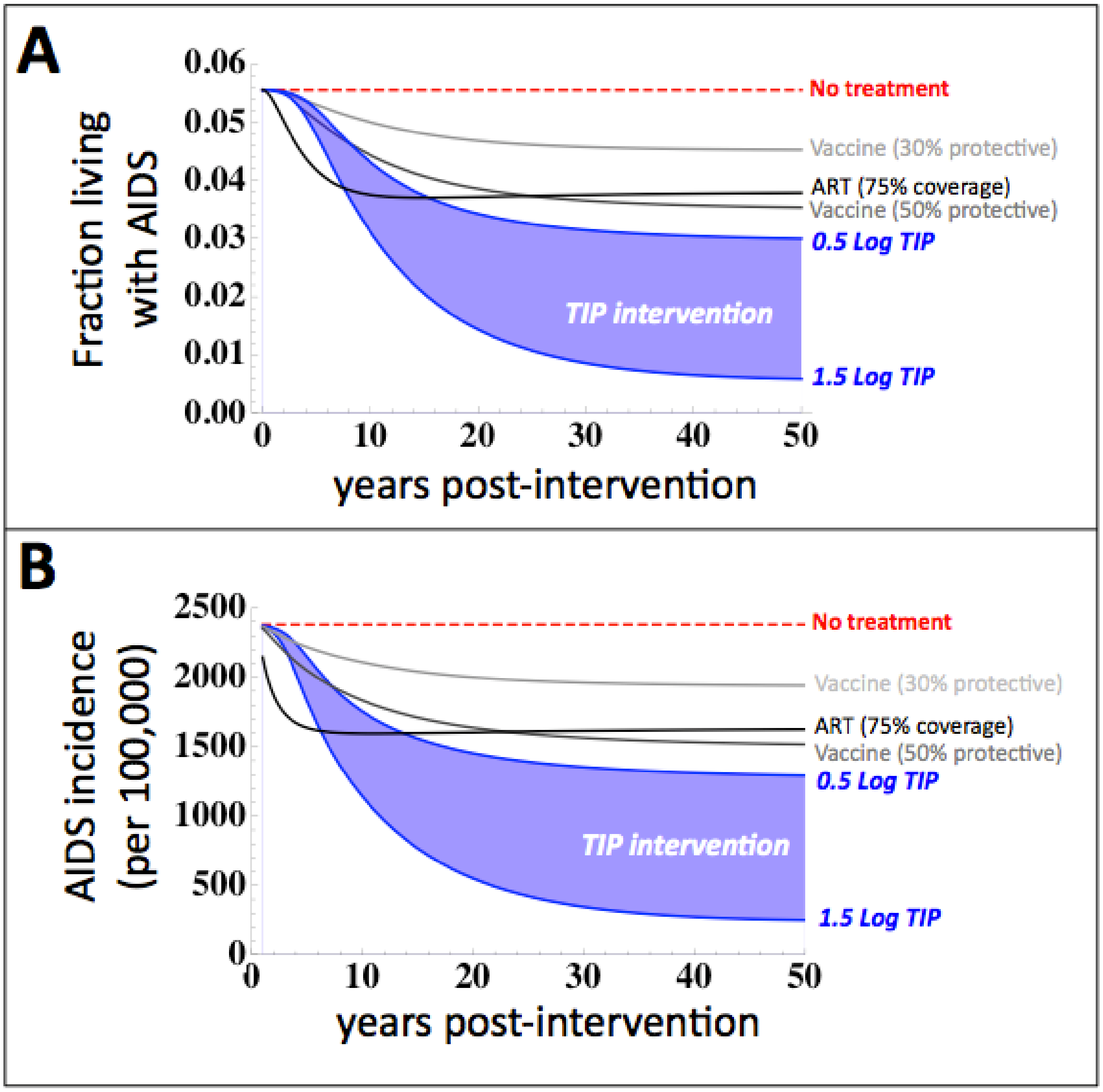

Supporting Figure S3: Projected impact of non-transmitting TIPs

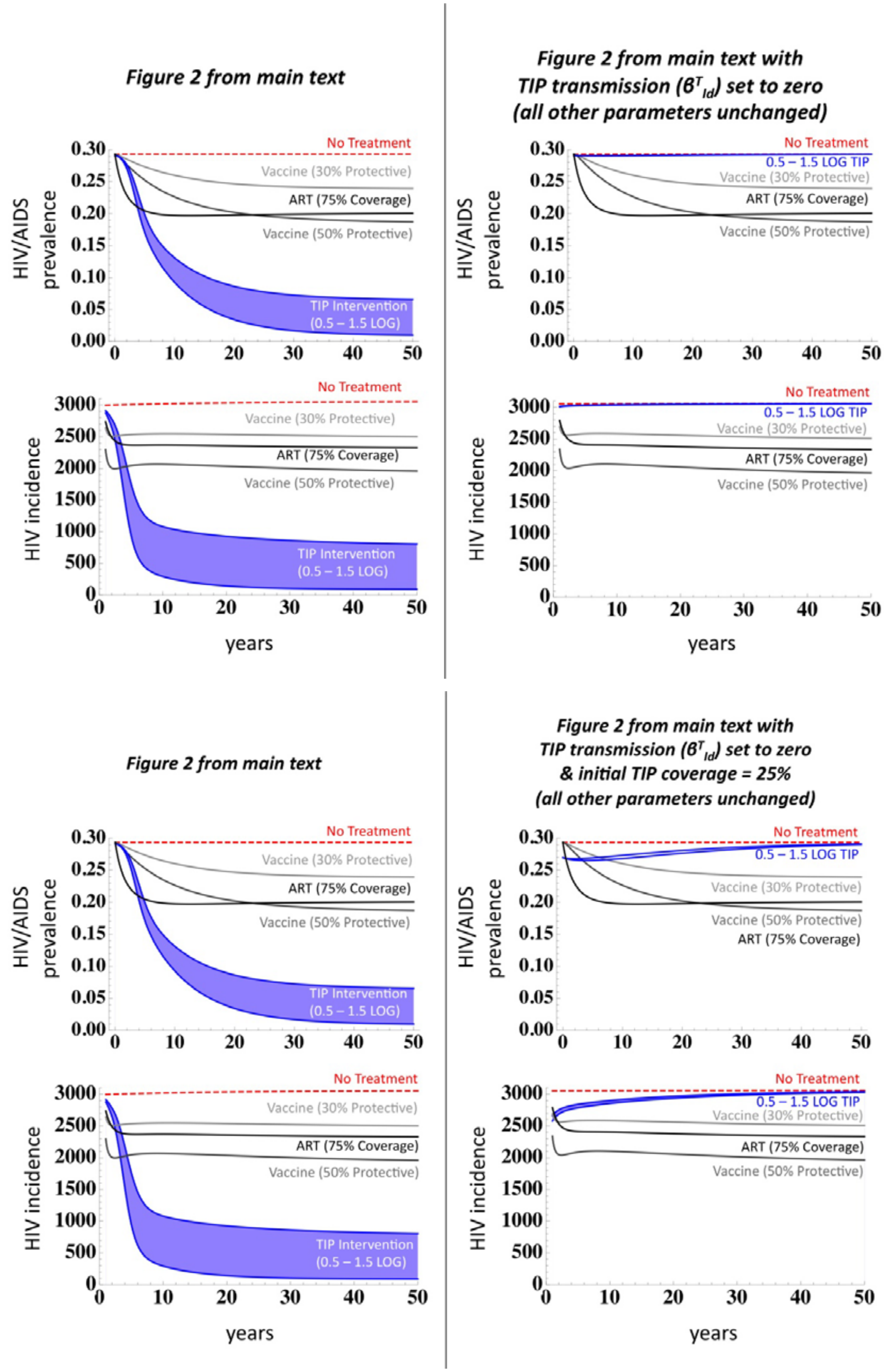

In the above figure, we compare the projected impact of TIP intervention to previous studies that have analyzed interventions that generate small reductions in patient viral load (e.g. the ~0.5-Log reduction in HIV viral load generated by acyclovir treatment [49]). Previous studies [49] projected that such a small reduction in viral load, due to acyclovir treatment for example, would have a minimal population-level impact on HIV/AIDS in sub-Saharan Africa. This stands in sharp contrast to our projections for TIP efficacy, but we emphasize that acyclovir is an ART-like pharmaceutical that does not transmit and thus is unable to rise in prevalence and autonomously target high-risk groups. To explore this difference of impact between TIP and acyclovir, we repeated simulations of TIP intervention but set TIP transmission between individuals to zero (i.e. we assumed that TIPs caused a 0.5-1.5 Log viral load reduction but did not transmit at all). The results demonstrate that a non-transmitting TIP has minimal impact on HIV prevalence or incidence, just as other authors found for acyclovir. Thus the increased efficacy of the TIP compared to other interventions (ART, vaccines, or acyclovir) is due to the unique ability of the TIP to be transmitted between individuals.

Sensitivity of the TIP R_0 (i.e. ability of TIP to stably propagate)

For TIP intervention to be effective, the TIP must stably propagate within individual patients and at the population scale. In order to examine the ability of the TIP to stably propagate, we examine the basic reproductive number, a standard measure of growth potential and 'invasibility' (i.e. whether a pathogen can increase in prevalence after introduction). The basic reproductive number (R_0) is the mean number of secondary infections caused by a typical infected individual in an otherwise completely susceptible population [11]. If $R_0 > 1$ for a pathogen, the pathogen infection can rise in prevalence in a susceptible population, and chronic infections such as HIV-1 will approach a stable steady-state; if $R_0 < 1$ the pathogen will die out. In this section, we derive approximate expressions for the basic reproductive number for TIP at the population level (R_{0pop}^{TIP}) and at the individual patient level ($R_{0in-vivo}^{TIP}$). We show that both R_{0pop}^{TIP} and $R_{0in-vivo}^{TIP}$ are greater than 1 for TIPs that reduce viral load between 0.5-Log and 1.5-Log.

Because propagation of the TIP depends on the existence of a wild-type HIV-1 epidemic, R_{0pop}^{TIP} is estimated in the context of an existing HIV-1 epidemic at steady-state based on a Susceptible-Infected-AIDS model.

We begin by addressing the basic reproductive number of HIV-1 and TIP in the individual patient *in vivo*. We previously characterized $R_{0in-vivo}^{TIP}$ [13] as:

$$R_{0in-vivo}^{TIP} = P^2 D(1 - \frac{1}{R_{0in-vivo}^{HIV}})$$

where $R_{0in-vivo}^{HIV}$ is the HIV-1 *in vivo* basic reproductive ratio

$$R_{0in-vivo}^{HIV} = \frac{bkn}{c\delta}$$

The *in vivo* basic reproductive number of HIV-1 is estimated to range between 3 and 11[11], which is similar to the value obtained using the parameters in Table S6.

In the current study, we created a new intracellular model that tracks the production of genomic mRNA and its subsequent packaging into virions by assuming that all other packaging materials are present in excess compared to genomic mRNA and that genomic mRNA is the limiting reagent for packaging. This new intracellular model introduces ρ and ψ in place of P and D from (10). Substituting ρ and ψ derived from the intracellular model in place of P and D yields a new expression for the *in vivo* basic reproductive number of a TIP:

$$R_{0in-vivo}^{TIP} = P^2 \frac{1-D}{1+P}(1 - \frac{1}{R_{0in-vivo}^{HIV}})$$

This $R_{0in-vivo}^{TIP}$ expression provides the criteria for intracellular properties of a TIP that will yield $R_{0in-vivo}^{TIP} > 1$, and hence allow for the design of an effective and sustained TIP intervention at the *in vivo* (within patient) level. For the parameters in Table S6:

Reduction in viral load predicted for TIP	$R_{0in-vivo}^{TIP}$
½ Log	2.23
1 Log	5.99
1 ½ Log	16.47

Thus, TIPs with the criteria used in our model stably propagate at the *in vivo* (within patient) level.

To estimate the basic reproductive number for both HIV-1 and a TIP at the population level, several simplifying assumptions are made. Since AIDS patients in the population model are more likely to die from AIDS than to infect a single partner, we ignore the minor effect of dually-infected AIDS patients (infected with both HIV-1 and TIP)

transmitting TIP in this derivation. For simplicity, the risk structure in the model is replaced by one SAC with c = 4.02 partners/year (the weighted average of the four SACs). Note that heterogeneity in contact rates is known to increase the reproductive number in a nonlinear fashion [50], so the estimates derived by this approach will be conservative.

First, we compute the population-level R_0 for wild-type HIV-1 in the basic Susceptible-Infected-AIDS model:

$$R_{0pop}^{HIV} = \frac{1}{\mu + \gamma_1} \beta_W^I c \frac{S}{N}$$

Here N represents the total sexually active population. Since the entire population is Susceptible, except for a single Infected individual, we make the approximation $N = S$. Therefore, the basic reproductive number for HIV-1 at the population level is given by:

$$R_{0pop}^{HIV} = \frac{\beta_W^I c}{\mu + \gamma_1}$$

Based on our chosen parameters,

$$R_{0pop}^{HIV} = \frac{\beta_W^I c}{\mu + \gamma_1} \approx 4.1$$

which is in rough agreement with measurements from field data [51]

Next we derive an approximate expression for R_{0pop}^{TIP}. As for R_{0pop}^{HIV}, which is estimated by considering one type of epidemiological event (the infection of Susceptible individuals with HIV-1), R_{0pop}^{TIP} is estimated by considering three types of

epidemiological events that directly increase the dually-infected population. Each of the following expressions describes the approximate number of events of each type that are expected to happen during the infectious lifetime of an I_d individual:

1) Infection of an I by an I_d in which TIP is transmitted:

$$\frac{1}{\mu+\gamma_2}\beta_T^{I_d} c \frac{I}{N}$$

2) Infection of an S by an I_d in which both TIP and HIV-1 transmission occur:

$$\frac{1}{\mu+\gamma_2}\beta_T^{I_d}\beta_W^{I_d} c \frac{S}{N}$$

3) Infection of an S by an I_d in which only TIP is transmitted (resulting in an S_t), followed by transmission of HIV-1 from an I to the S_t:

$$\frac{1}{\mu+\gamma_2}\beta_T^{I_d}(1-\beta_W^{I_d})c\frac{S}{N}(\frac{1}{\mu}\beta_W^{I} c\frac{I}{N})$$

These three transmission events are the major contributing epidemiological events that increase the I_d population and therefore contribute to R_{0pop}^{TIP}.

Infection of an S by an I_d in which TIP is transmitted (generating an S_t), followed by transmission of HIV-1 originating from an I_d can be ignored because this mechanism depends on two separate contacts with an I_d individual. Because the basic reproductive number for the TIP pertains to the situation that exists at the time of TIP introduction, when dually-infected (I_d) individuals are extremely rare ($I_d = 1$), we make the simplifying approximation:

$$\frac{I_d}{N}\approx 0$$

Considering these three major contributions to the growth of the I_d populations yields:

$$R_{0pop}^{TIP} \approx \frac{1}{\mu+\gamma_2}\left(\beta_T^{I_d} c\frac{I}{N} + \beta_T^{I_d}\beta_W^{I_d} c\frac{S}{N} + \beta_T^{I_d}(1-\beta_W^{I_d})c\frac{S}{N}(\frac{1}{\mu}\beta_W^{I} c\frac{I}{N})\right)$$

and upon algebraic simplification:

$$R_{0pop}^{TIP} \approx \beta_T^{I_d}\frac{1}{\mu+\gamma_2}\left(c\frac{I}{N} + \beta_W^{I_d} c\frac{S}{N} + (1-\beta_W^{I_d})c\frac{S}{N}(\frac{1}{\mu}\beta_W^{I} c\frac{I}{N})\right)$$

This equation can be further simplified by substituting in steady-state values for *S* and *I* from the basic Susceptible-Infected-AIDS model and then rewritten in terms of R_{0pop}^{HIV} to yield:

$$R_{0pop}^{TIP} = \frac{c\beta_T^{I_d}\left[(R_{0pop}^{HIV}-1)\gamma_1(\beta_W^{I_d}-1) + R_{0pop}^{HIV}\beta_W^{I_d}\mu\right]}{R_{0pop}^{HIV}\mu(\gamma_2+\mu)}$$

This expression is dependent upon the R_{0pop}^{HIV}, and substituting in parameter values from Table S1 shows that R_{0pop}^{TIP} is always substantially greater than 1 for the parameters in our model.

Reduction in viral load predicted for TIP	R_{0pop}^{TIP}
½ Log	8.73
1 Log	11.86
1 ½ Log	16.15

The above analysis neglects risk structure, which can drastically lower the parameter thresholds at which $R_0 > 1$[52]. Indeed, in the full TIP model (containing risk structure), all TIPs we tested (0.5 Log – 1.5 Log) are able to stably propagate at the population level for all parameter regimes where HIV-1 is stably propagating.

Parameter Sensitivity Analysis

Figure 5 (main text) is essentially a parameter sensitivity analysis for the two most relevant parameters from the single-cell level model. Here, we expand this sensitivity analysis to examine HIV-1/AIDS prevalence for other relevant parameters.

Sensitivity Analysis as a function of changes in risk-structure

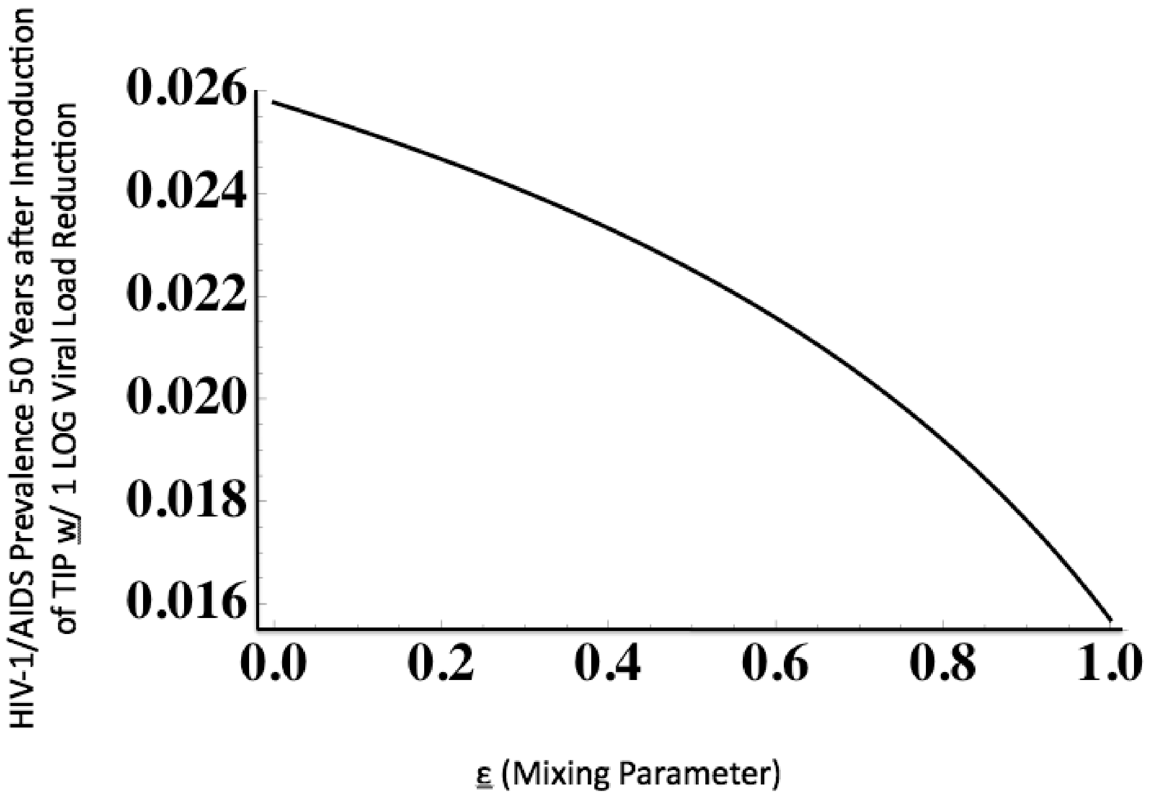

Sensitivity Analysis as a function of changes in population removal (death)

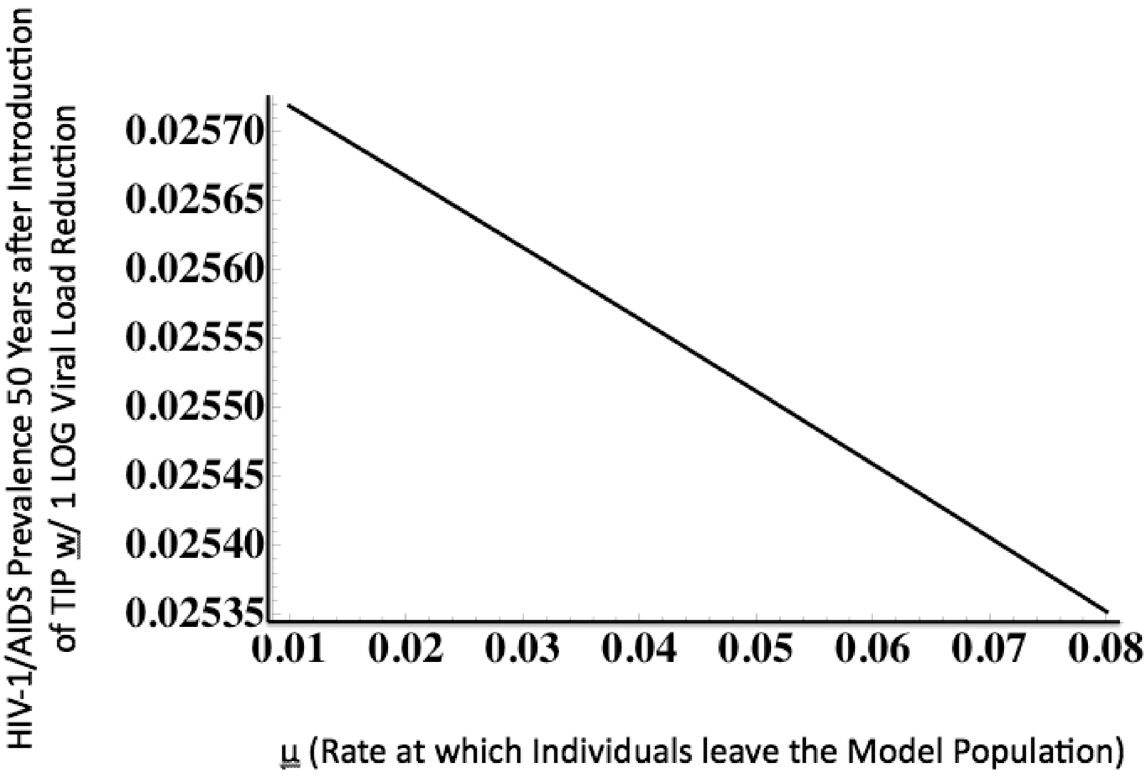

Sensitivity Analysis of HIV-1 prevalence as a function of AIDS death rate

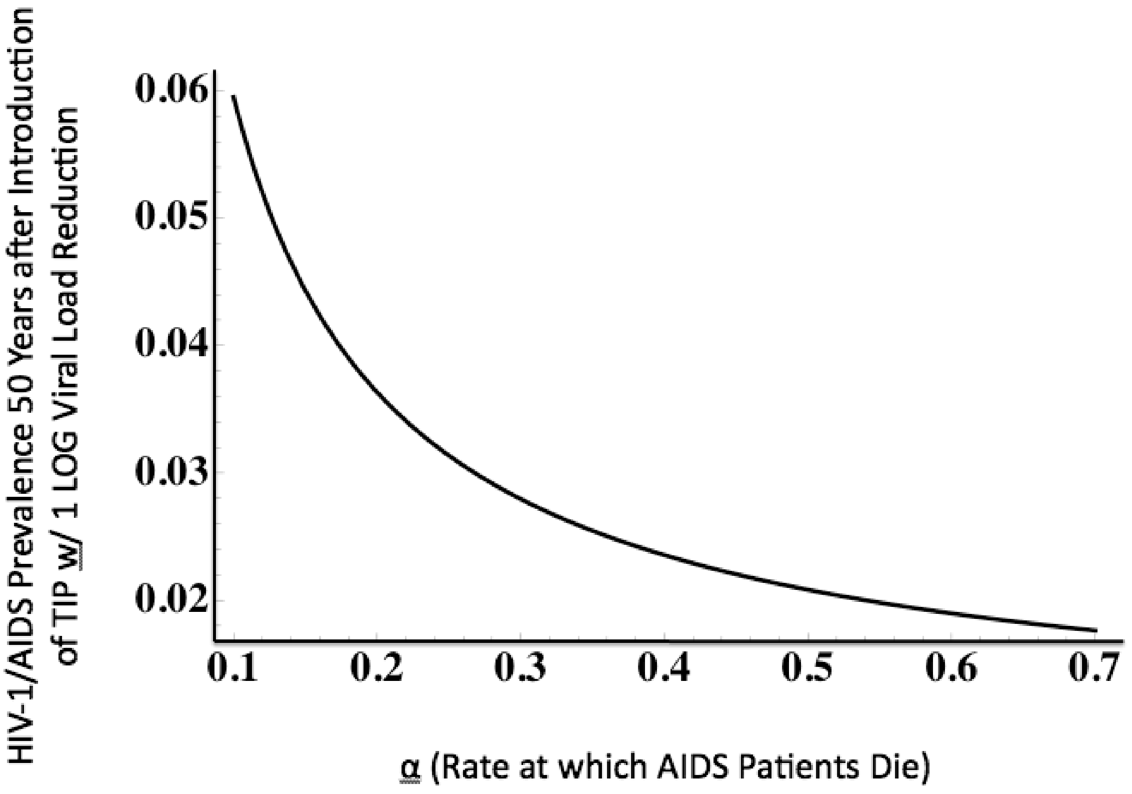

Finally, we also expand the sensitivity analysis of Figure 5 (main text) to examine HIV-1/AIDS prevalence in the event of TIP upregulation of HIV-1 as well as in the presence of behavioral disinhibition.

Sensitivity of HIV-1/AIDS prevalence to TIP Genome Expression (P) and Fraction Upregulation/Inhibition of HIV-1 (D)

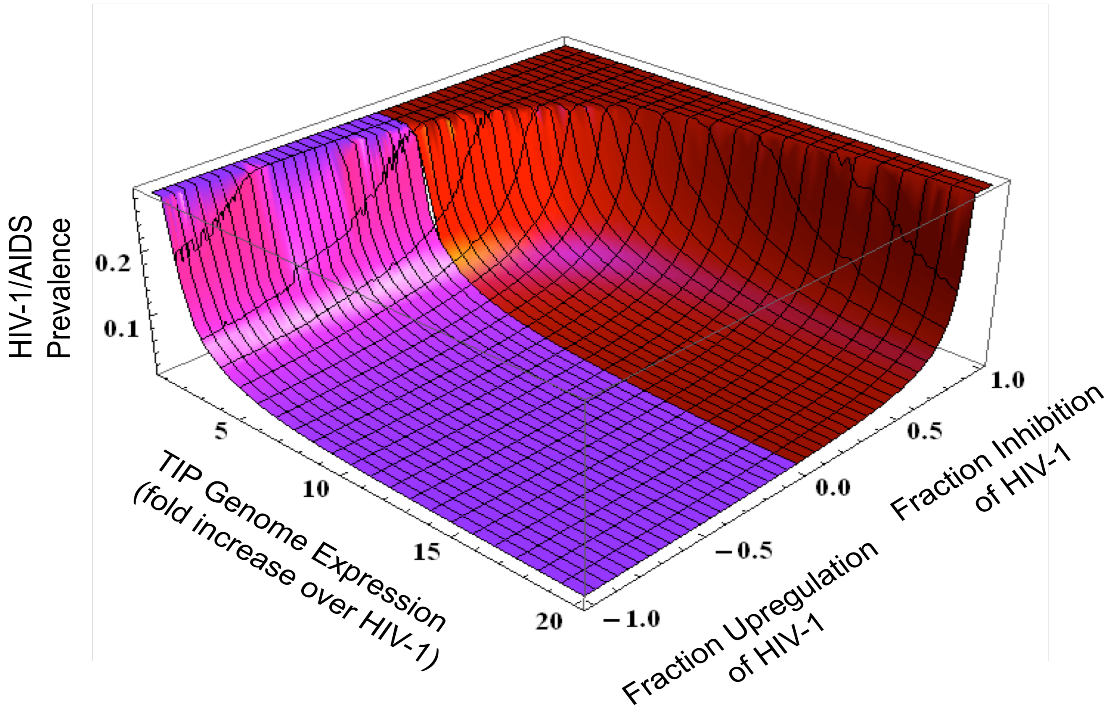

Sensitivity of HIV-1/AIDS Prevalence to changes in TIP Genome Expression (*P*) and Fraction Upregulation/Inhibition of HIV-1 (*D*) with Behavioral Disinhibition

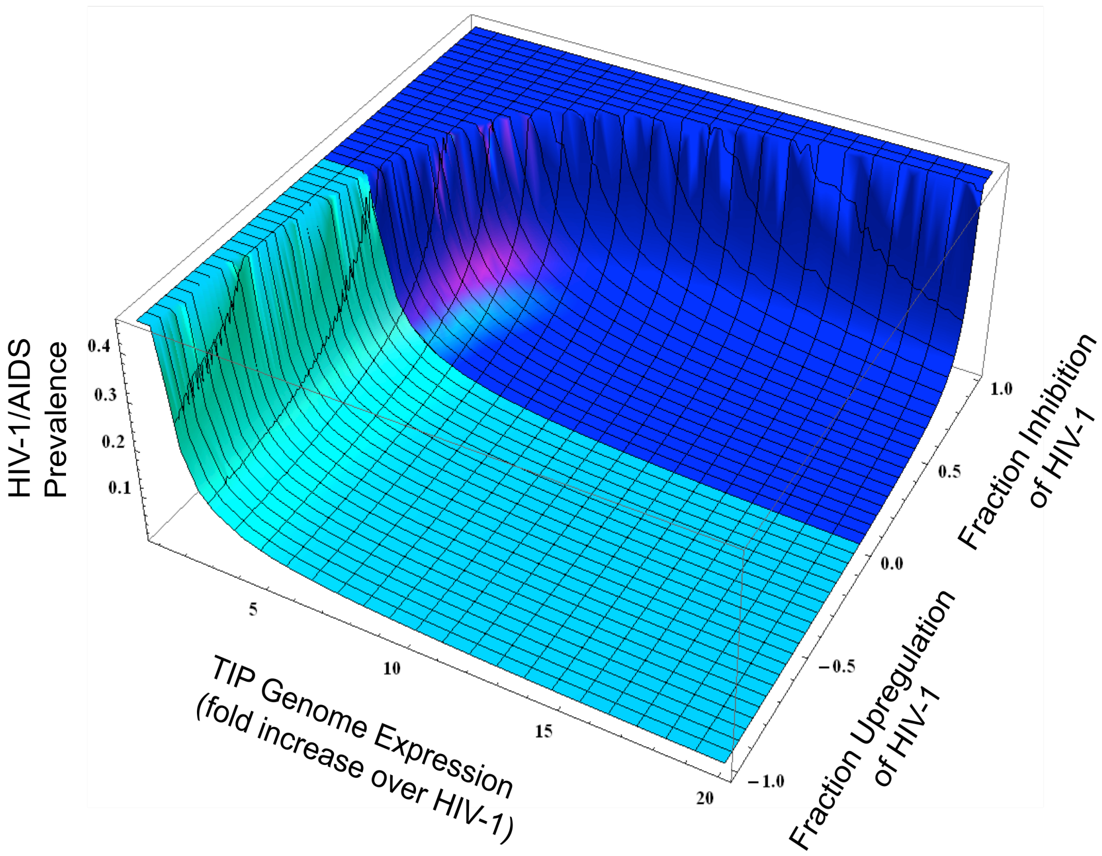

Sensitivity of model when transmission function is re-scaled

Our main analyses of the TIP intervention were based on a rescaled version of the transmission-vs-viral load set-point function reported by Fraser et al. [9], where the rescaling was motivated by the need to reconcile the functional form reported by Fraser with the population context of the Baggaley model. Here we study the sensitivity of our projections for TIP intervention to the assumed relationship between the viral-load and transmission probability, by considering an alternate parameterization of the viral-load transmission curve.

Our initial rescaling used a maximum per-partnership transmission probability of 0.54, and a mean probability of 0.105 per partnership at the mean viral load of 100,000 copies/mL, to correspond with the assumptions of [1] (see supporting information page 24). This leads to a function that saturates near 10^7 copies/mL and has its half-saturation point at 414,300 copies/mL (see figure below). The alternate curve saturates has its maximum per-partnership transmission probability set to 0.127, and its half-saturation point is 20,518 copies/mL. As a result, the curve saturates near 10^6 copies/mL and the mean viral load of 100,000 copies/ml is no longer positioned at the steepest section of the viral-load transmission curve and is instead much closer to the saturation point:

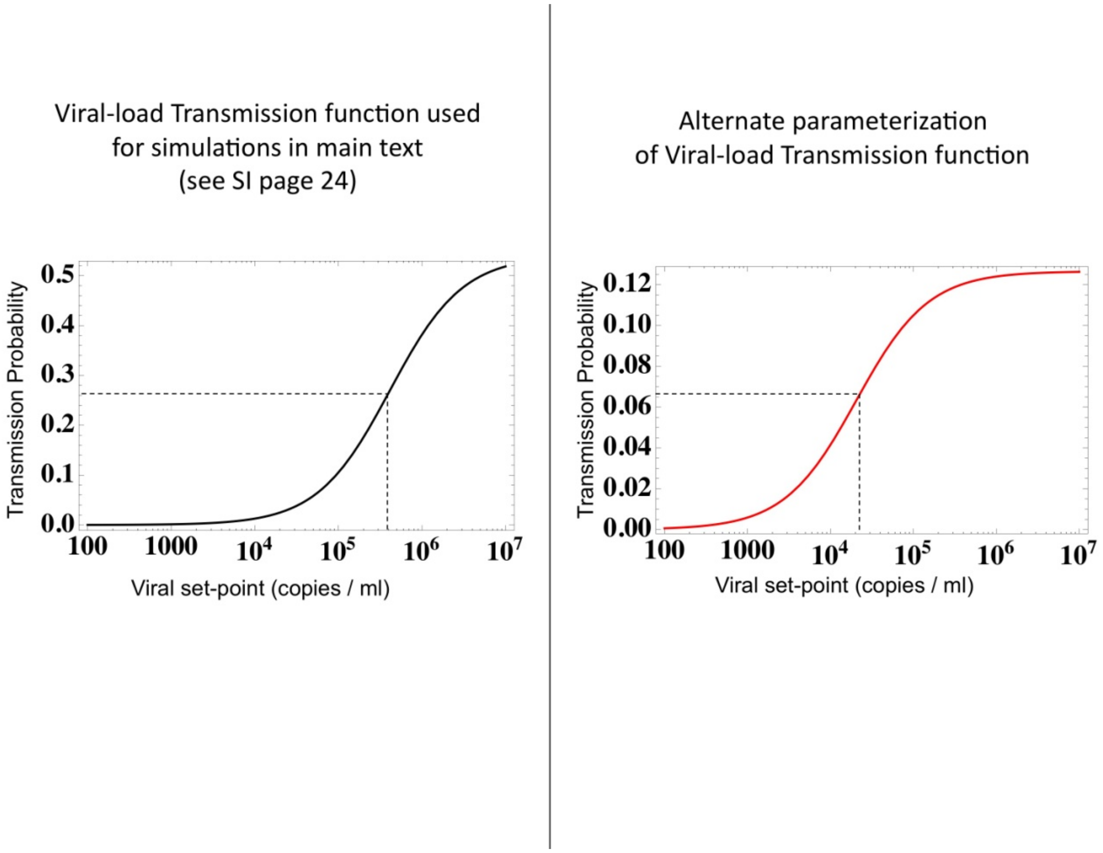

Repeating the simulations of TIP intervention using this alternate parameterization of the Fraser viral-load transmission function generates qualitatively similar projections:

Figure 2, main text (using viral-load transmission function From page 24)

Figure 2 simulations repeated using Alternate viral-load transmission function (all other parameters remain unchanged)

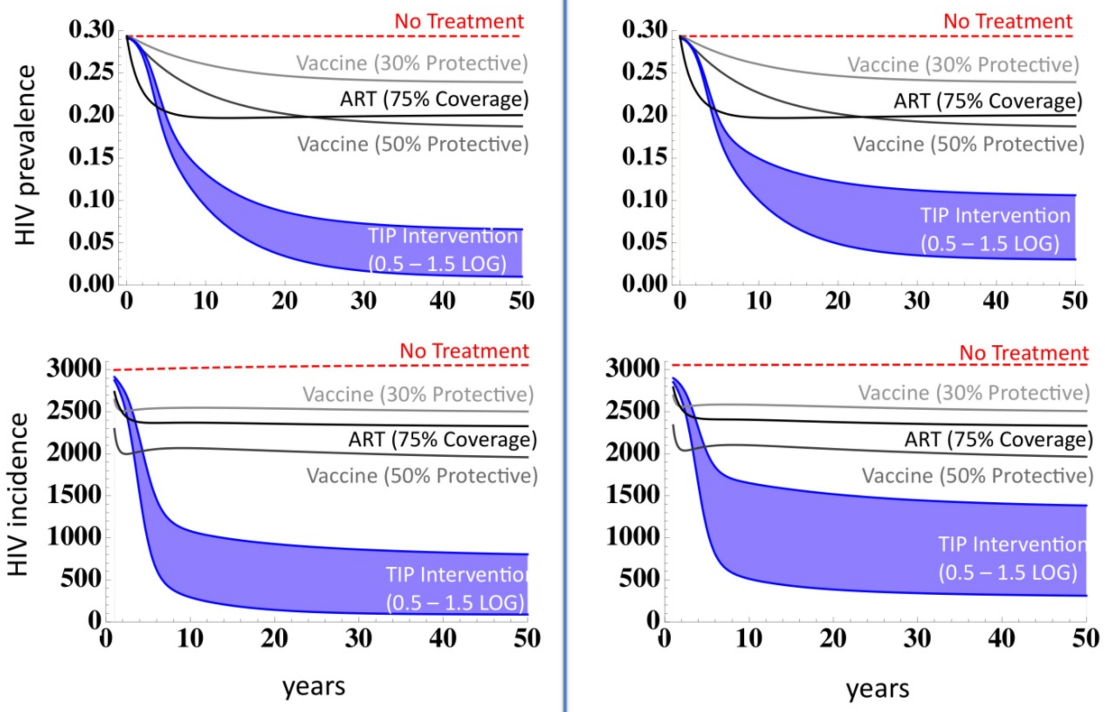

Sensitivity of Model if TIP-infected individuals (S_T) revert to susceptible (S)

The purpose of this analysis is to determine the sensitivity of TIP reversion (loss of TIP in S_T individuals) to the predicted efficacy of a TIP intervention at the population scale. TIPs integrate and thus are likely to enter long-lived CD4 memory cells. The formation of HIV-1 latent reservoirs has been observed to occur during the first week of infection [53]. Gene therapy studies reveal that cells with integrated therapy vectors (even peripheral B cells) are maintained for years but these therapies use a large injection of transduced cells [28]. We explore the sensitivity of TIP reversion by modifying the TIP Population model to account for S_T individuals losing the TIP (and thus becoming reclassified as S individuals) at the rate μ_s. In the figures below, we explore S_T reversion occurring with a half-life of 6 months, 1 month, 1 week, and 1 day:

Figure 2 from main text
No $S_T \rightarrow S$ ($\mu_s \approx 0$/year)
(TIP trajectories only)

Figure 2 from main text with
$S_T \rightarrow S$ set to 6 month half-life ($\mu_s \approx 1.38$/year)
(all other parameters unchanged)

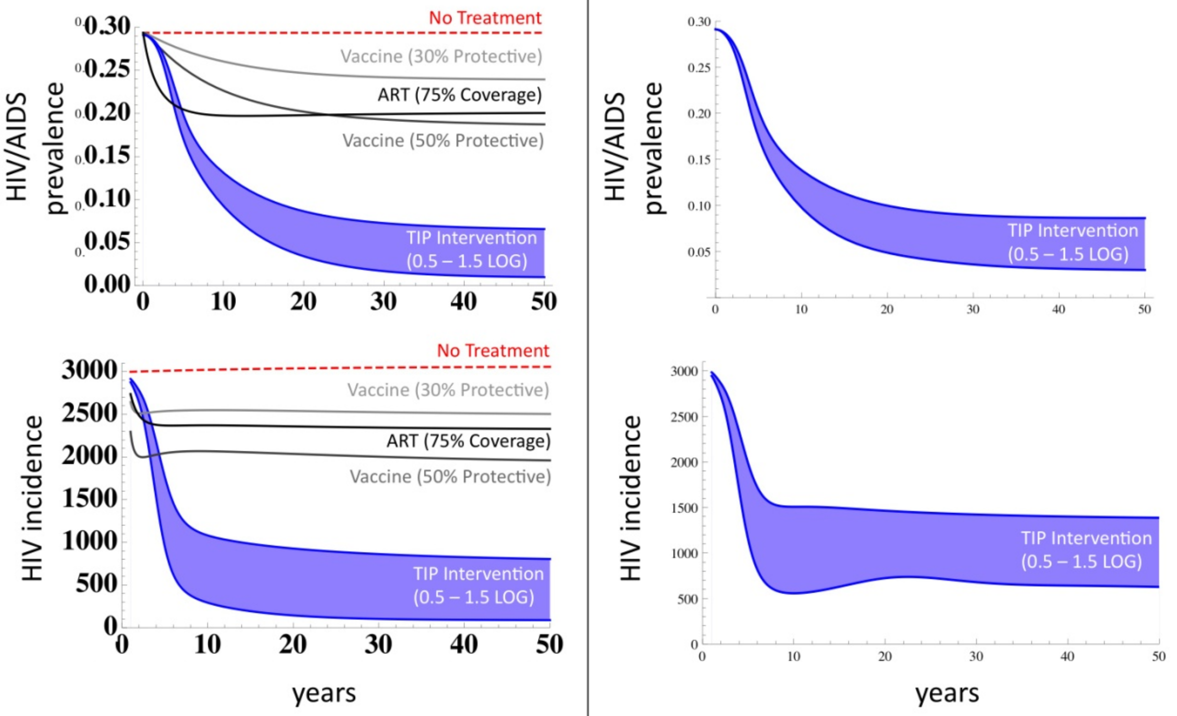

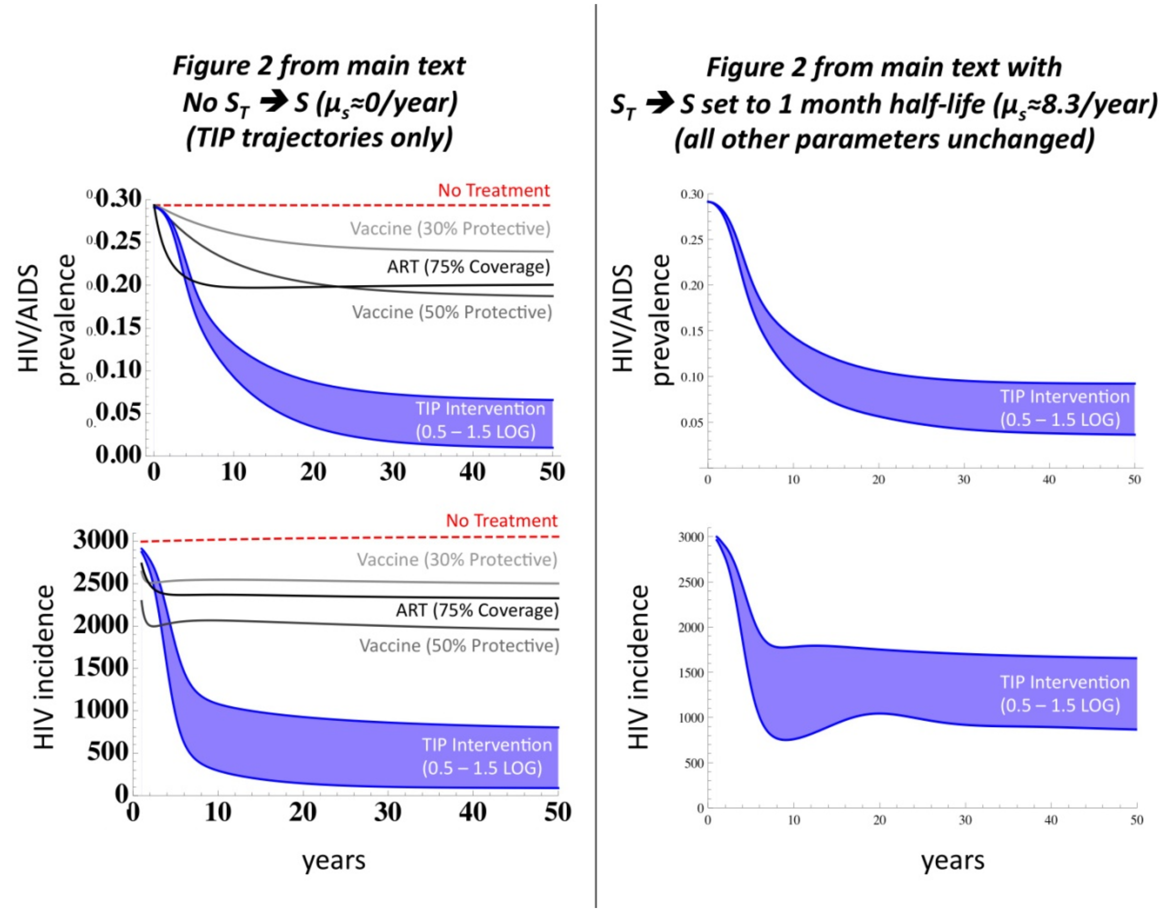
Figure 2 from main text
No S_T ➔ S (μ_s≈0/year)
(TIP trajectories only)
Figure 2 from main text with
S_T ➔ S set to 1 month half-life (μ_s≈8.3/year)
(all other parameters unchanged)
No Treatment
Vaccine (30% Protective)
ART (75% Coverage)
Vaccine (50% Protective)
TIP Intervention
(0.5 – 1.5 LOG)
HIV/AIDS prevalence
HIV incidence
years

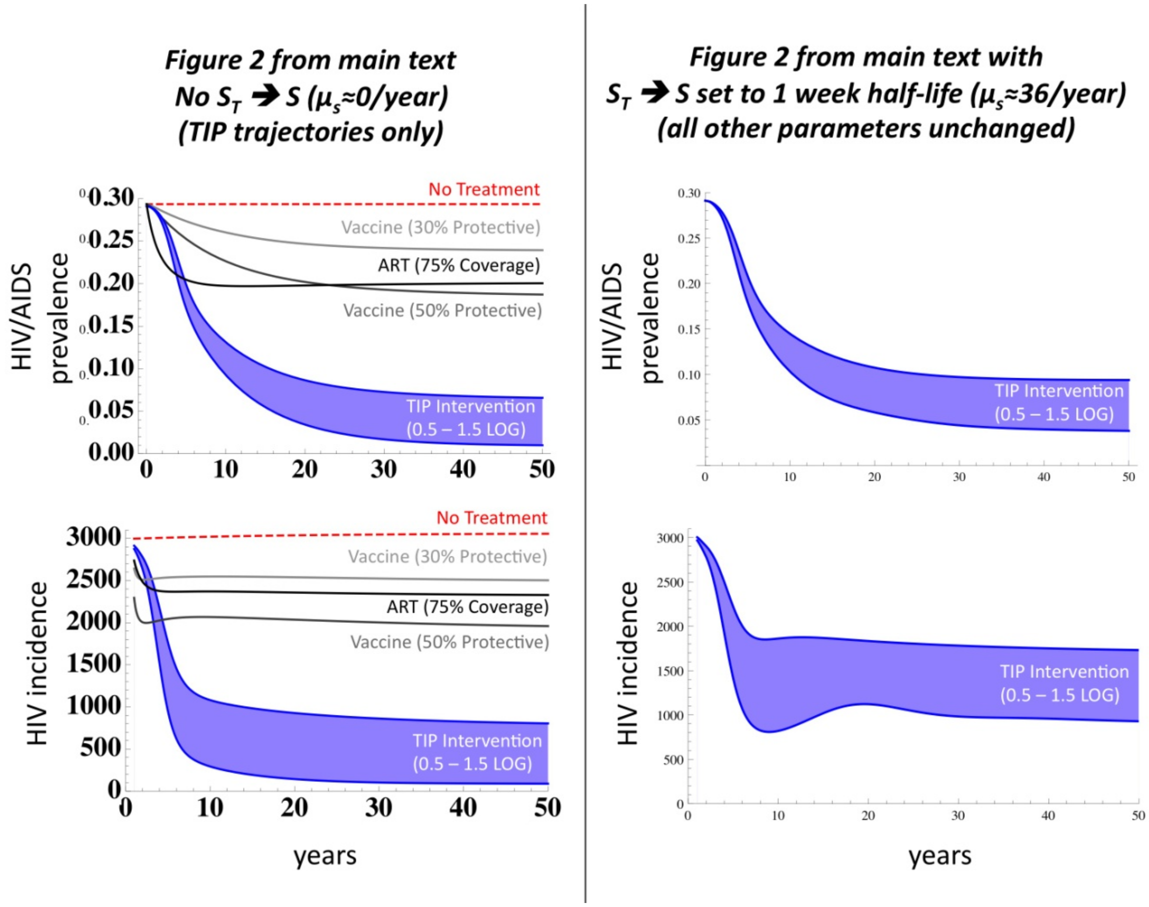
Figure 2 from main text
No S_T ➔ S (μ_s≈0/year)
(TIP trajectories only)
Figure 2 from main text with
S_T ➔ S set to 1 week half-life (μ_s≈36/year)
(all other parameters unchanged)
HIV/AIDS prevalence
No Treatment
Vaccine (30% Protective)
ART (75% Coverage)
Vaccine (50% Protective)
TIP Intervention
(0.5 – 1.5 LOG)
HIV incidence
years

Figure 2 from main text
No S_T ➔ S (μ_s≈0/year)
(TIP trajectories only)

Figure 2 from main text with
S_T ➔ S set to 1 day half-life (μ_s≈253/year)
(all other parameters unchanged)

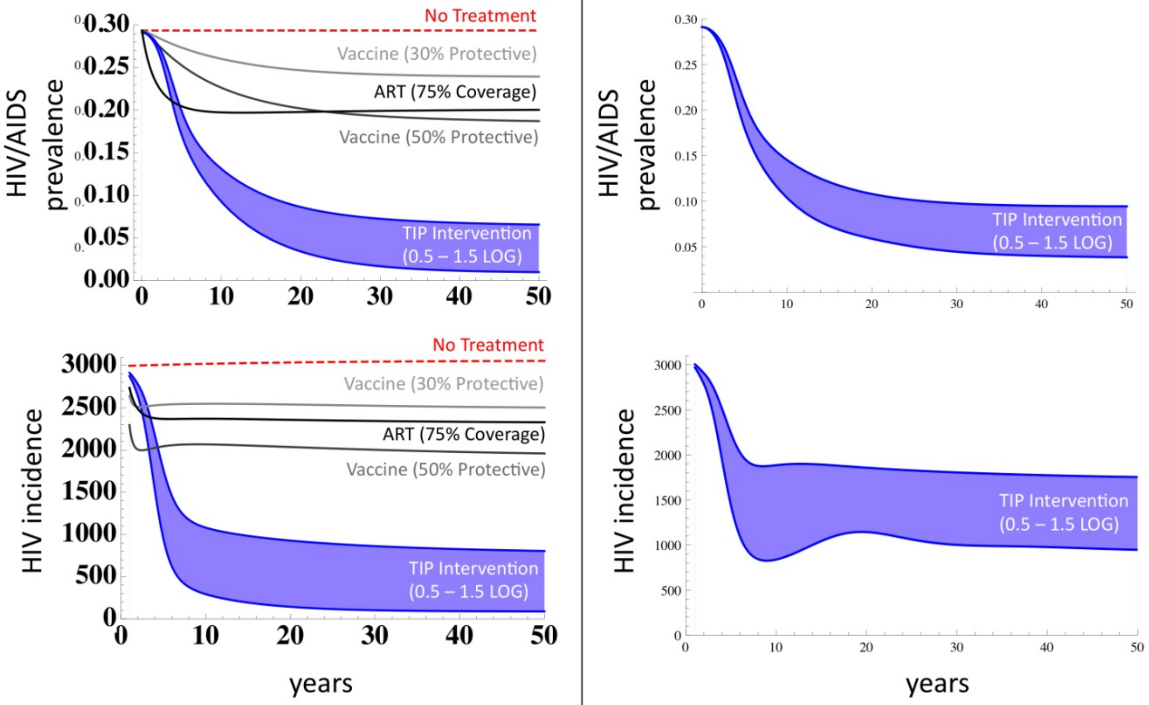

Sensitivity of model to removal of independent transmission of TIPs (i.e. removal of S_T individuals)

As an extreme sensitivity analysis of our assumptions about TIP transmission, we consider the case where TIPs cannot be transmitted independently to susceptible individuals. We removed the class of individuals who are infected with only TIP (S_T individuals) so that TIPs can only spread to individuals already infected with HIV, or else simultaneously with HIV. We achieved this effect within the current model structure by making the S_T class revert back to S at a rate of 10^6 per year, so that the S_T population was effectively zero. Despite the elimination of the S_T class of individuals, TIPs are still predicted to have a significant qualitative effect of reducing disease prevalence at the population level:

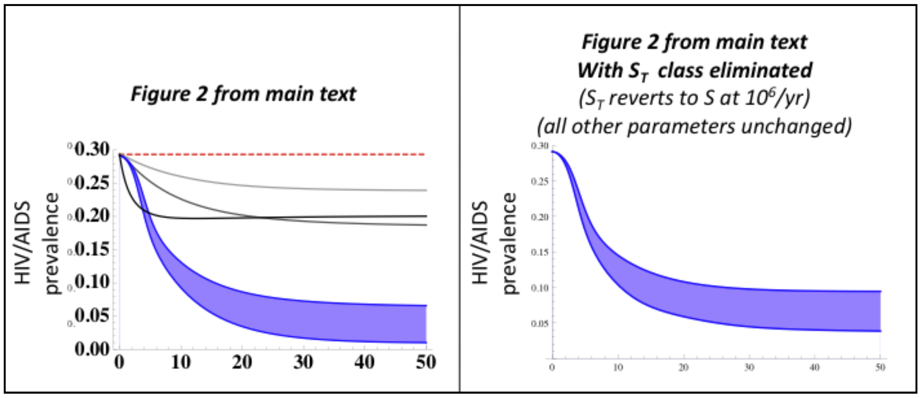

This robust effect is due to the TIPs following the same transmission route as wild-type HIV-1 and autonomously targeting high-risk individuals. Because of their high contact rates, these individuals are infected rapidly by HIV-1 and subsequently by TIPs. These high-risk individuals are responsible for the majority of HIV-1 disease transmission in uncontrolled populations, so the targeting of this group increases the efficacy of TIPs at the population level.

Supporting References

1. Baggaley RF, Garnett GP, Ferguson NM (2006) Modelling the impact of antiretroviral use in resource-poor settings. PLoS Med 3: e124.
2. Blower SM, McLean AR (1994) Prophylactic vaccines, risk behavior change, and the probability of eradicating HIV in San Francisco. Science 265: 1451-1454.
3. Korber B, Muldoon M, Theiler J, Gao F, Gupta R, et al. (2000) Timing the ancestor of the HIV-1 pandemic strains. Science 288: 1789-1796.
4. Granich RM, Gilks CF, Dye C, De Cock KM, Williams BG (2009) Universal voluntary HIV testing with immediate antiretroviral therapy as a strategy for elimination of HIV transmission: a mathematical model. Lancet 373: 48-57.
5. Rosen S, Sanne I, Collier A, Simon JL (2005) Rationing antiretroviral therapy for HIV/AIDS in Africa: Choices and consequences. Plos Medicine 2: 1098-1104.
6. Rosen S, Fox MP, Gill CJ (2007) Patient retention in antiretroviral therapy programs in sub-Saharan Africa: A systematic review. Plos Medicine 4: 1691-1701.
7. Fox MP, Rosen S (2010) Patient retention in antiretroviral therapy programs up to three years on treatment in sub-Saharan Africa, 2007-2009: systematic review. Tropical Medicine & International Health 15: 1-15.
8. Donnell D, Baeten JM, Kiarie J, Thomas KK, Stevens W, et al. Heterosexual HIV-1 transmission after initiation of antiretroviral therapy: a prospective cohort analysis. Lancet 375: 2092-2098.
9. Fraser C, Hollingsworth TD, Chapman R, de Wolf F, Hanage WP (2007) Variation in HIV-1 set-point viral load: epidemiological analysis and an evolutionary hypothesis. Proc Natl Acad Sci U S A 104: 17441-17446.
10. Mellors JW, Rinaldo CR, Jr., Gupta P, White RM, Todd JA, et al. (1996) Prognosis in HIV-1 infection predicted by the quantity of virus in plasma. Science 272: 1167-1170.
11. Nowak MA, May RM (2000) Virus dynamics : mathematical principles of immunology and virology. Oxford ; New York: Oxford University Press. xii, 237 p. p.
12. Perelson AS, Nelson PW (1999) Mathematical analysis of HIV-1 dynamics in vivo. Siam Review 41: 3-44.
13. Weinberger LS, Schaffer DV, Arkin AP (2003) Theoretical design of a gene therapy to prevent AIDS but not human immunodeficiency virus type 1 infection. J Virol 77: 10028-10036.
14. Chen J, Nikolaitchik O, Singh J, Wright A, Bencsics CE, et al. (2009) High efficiency of HIV-1 genomic RNA packaging and heterozygote formation revealed by single virion analysis. Proc Natl Acad Sci U S A 106: 13535-13540.
15. Holland JJ (1990) Generation and replication of defective viral genomes. In: Fields BN, Knipe DM, editors. Fields Virology. 2nd ed. New York: Raven Press. pp. 77-99.

16. Houzet L, Paillart JC, Smagulova F, Maurel S, Morichaud Z, et al. (2007) HIV controls the selective packaging of genomic, spliced viral and cellular RNAs into virions through different mechanisms. Nucleic Acids Research 35: 2695-2704.
17. Whitney JB, Wainberg MA (2006) Impaired RNA incorporation and dimerization in live attenuated leader-variants of SIVmac239. Retrovirology 3: -.
18. Purcell DFJ, Martin MA (1993) Alternative Splicing of Human-Immunodeficiency-Virus Type-1 Messenger-Rna Modulates Viral Protein Expression, Replication, and Infectivity. Journal of Virology 67: 6365-6378.
19. Koldej RM, Anson DS (2009) Refinement of lentiviral vector for improved RNA processing and reduced rates of self inactivation repair. Bmc Biotechnology 9: -.
20. Bohne J, Wodrich H, Krausslich HG (2005) Splicing of human immunodeficiency virus RNA is position-dependent suggesting sequential removal of introns from the 5 ' end. Nucleic Acids Research 33: 825-837.
21. Purcell DF, Martin MA (1993) Alternative splicing of human immunodeficiency virus type 1 mRNA modulates viral protein expression, replication, and infectivity. J Virol 67: 6365-6378.
22. Keele BF, Giorgi EE, Salazar-Gonzalez JF, Decker JM, Pham KT, et al. (2008) Identification and characterization of transmitted and early founder virus envelopes in primary HIV-1 infection. Proc Natl Acad Sci U S A 105: 7552-7557.
23. Learn GH, Muthui D, Brodie SJ, Zhu T, Diem K, et al. (2002) Virus population homogenization following acute human immunodeficiency virus type 1 infection. J Virol 76: 11953-11959.
24. Haaland RE, Hawkins PA, Salazar-Gonzalez J, Johnson A, Tichacek A, et al. (2009) Inflammatory genital infections mitigate a severe genetic bottleneck in heterosexual transmission of subtype A and C HIV-1. PLoS Pathog 5: e1000274.
25. Salazar-Gonzalez JF, Bailes E, Pham KT, Salazar MG, Guffey MB, et al. (2008) Deciphering human immunodeficiency virus type 1 transmission and early envelope diversification by single-genome amplification and sequencing. J Virol 82: 3952-3970.
26. Miller CJ, Li Q, Abel K, Kim EY, Ma ZM, et al. (2005) Propagation and dissemination of infection after vaginal transmission of simian immunodeficiency virus. J Virol 79: 9217-9227.
27. Zhang Z, Schuler T, Zupancic M, Wietgrefe S, Staskus KA, et al. (1999) Sexual transmission and propagation of SIV and HIV in resting and activated CD4+ T cells. Science 286: 1353-1357.
28. Bordignon C, Notarangelo LD, Nobili N, Ferrari G, Casorati G, et al. (1995) Gene therapy in peripheral blood lymphocytes and bone marrow for ADA-immunodeficient patients. Science 270: 470-475.
29. Levine BL, Humeau LM, Boyer J, MacGregor RR, Rebello T, et al. (2006) Gene transfer in humans using a conditionally replicating lentiviral vector. Proc Natl Acad Sci U S A 103: 17372-17377.

30. Kuritzkes DR, D.Walker B (2007) HIV-1 Pathogenesis, Clinical Manifestations and Treatment. In: Knipe DM, editor. Field's Virology. 5 ed. Philadelphia: Lippincott Williams & Wilkins.
31. Wooley DP, Smith RA, Czajak S, Desrosiers RC (1997) Direct demonstration of retroviral recombination in a rhesus monkey. J Virol 71: 9650-9653.
32. Kim EY, Busch M, Abel K, Fritts L, Bustamante P, et al. (2005) Retroviral recombination in vivo: viral replication patterns and genetic structure of simian immunodeficiency virus (SIV) populations in rhesus macaques after simultaneous or sequential intravaginal inoculation with SIVmac239Deltavpx/Deltavpr and SIVmac239Deltanef. J Virol 79: 4886-4895.
33. Porco TC, Blower SM (1998) Designing HIV vaccination policies: Subtypes and cross-immunity. Interfaces 28: 167-190.
34. McLean AR, Blower SM (1993) Imperfect vaccines and herd immunity to HIV. Proc Biol Sci 253: 9-13.
35. Enterprise GHV (2009).
36. McLean AR, Blower SM (1995) Modelling HIV vaccination. Trends Microbiol 3: 458-462.
37. Rerks-Ngarm S, Pitisuttithum P, Nitayaphan S, Kaewkungwal J, Chiu J, et al. (2009) Vaccination with ALVAC and AIDSVAX to prevent HIV-1 infection in Thailand. N Engl J Med 361: 2209-2220.
38. Phillips AN (1996) Reduction of HIV concentration during acute infection: independence from a specific immune response. Science 271: 497-499.
39. Mohri H, Perelson AS, Tung K, Ribeiro RM, Ramratnam B, et al. (2001) Increased turnover of T lymphocytes in HIV-1 infection and its reduction by antiretroviral therapy. J Exp Med 194: 1277-1287.
40. Wei XP, Ghosh SK, Taylor ME, Johnson VA, Emini EA, et al. (1995) Viral Dynamics in Human-Immunodeficiency-Virus Type-1 Infection. Nature 373: 117-122.
41. Wei X, Ghosh SK, Taylor ME, Johnson VA, Emini EA, et al. (1995) Viral dynamics in human immunodeficiency virus type 1 infection. Nature 373: 117-122.
42. Haase AT, Henry K, Zupancic M, Sedgewick G, Faust RA, et al. (1996) Quantitative image analysis of HIV-1 infection in lymphoid tissue. Science 274: 985-989.
43. Ramratnam B, Bonhoeffer S, Binley J, Hurley A, Zhang L, et al. (1999) Rapid production and clearance of HIV-1 and hepatitis C virus assessed by large volume plasma apheresis. Lancet 354: 1782-1785.
44. Reddy B, Yin J (1999) Quantitative intracellular kinetics of HIV type 1. AIDS Res Hum Retroviruses 15: 273-283.
45. Kim H, Yin J (2005) Robust growth of human immunodeficiency virus type 1 (HIV-1). Biophys J 89: 2210-2221.
46. Okamoto T, Wong-Staal F (1986) Demonstration of virus-specific transcriptional activator(s) in cells infected with HTLV-III by an in vitro cell-free system. Cell 47: 29-35.
47. Weinberger LS, Dar RD, Simpson ML (2008) Transient-mediated fate determination in a transcriptional circuit of HIV. Nat Genet 40: 466-470.
48. Jouvenet N, Bieniasz PD, Simon SM (2008) Imaging the biogenesis of individual HIV-1 virions in live cells. Nature 454: 236-240.

49. Baggaley RF, Griffin JT, Chapman R, Hollingsworth TD, Nagot N, et al. (2009) Estimating the public health impact of the effect of herpes simplex virus suppressive therapy on plasma HIV-1 viral load. AIDS 23: 1005-1013.
50. Anderson RM, Gupta S, May RM (1991) Potential of community-wide chemotherapy or immunotherapy to control the spread of HIV-1. Nature 350: 356-359.
51. Anderson RM, May RM (1988) Epidemiological parameters of HIV transmission. Nature 333: 514-519.
52. Anderson R (1991) Infectious diseases of humans: dynamics and control: Oxford University Press, Oxford; New York.
53. Han Y, Wind-Rotolo M, Yang HC, Siliciano JD, Siliciano RF (2007) Experimental approaches to the study of HIV-1 latency. Nat Rev Microbiol 5: 95-106.
